# Hyperuricemia risk in bempedoic acid-treated hyperlipidemic patients

**DOI:** 10.1038/s43856-026-01545-2

**Published:** 2026-04-01

**Authors:** Po-Hsueh Su, Daniel Hsiang-Te Tsai, Miyuki Hsing-Chun Hsieh, Tharmaraj Vairaperumal, Edward Chia-Cheng Lai, Ping-Yen Liu

**Affiliations:** 1 From the Division of Cardiology, Department of Internal Medicine, National Cheng Kung University Hospital, College of Medicine, National Cheng Kung University, Tainan, Taiwan; 2 Division of Cardiology, Department of Internal Medicine, Madou Sin-Lau Hospital, Tainan, Taiwan; 3 School of Pharmacy, Institute of Clinical Pharmacy and Pharmaceutical Sciences, College of Medicine, National Cheng Kung University, Tainan, Taiwan; 4 Population Health Data Center, National Cheng Kung University, Tainan, Taiwan; 5 Institute of Clinical Medicine, College of Medicine, National Cheng Kung University, Tainan, Taiwan

**Keywords:** Dyslipidaemias, Renovascular hypertension

## Abstract

**Background:**

Bempedoic acid is an oral cholesterol-lowering medication used to reduce low-density lipoprotein cholesterol (LDL-C), particularly in patients who require additional lipid lowering. Clinical trials have shown that bempedoic acid increases serum uric acid levels, but the implications of this effect in real-world clinical practice, including the need for anti-gout treatment, are not well defined.

**Methods:**

We conducted a retrospective cohort study using a large, multi-institutional U.S. electronic health record database. Adult patients with hyperlipidemia who have newly initiated bempedoic acid were compared with those initiating ezetimibe. Propensity score matching was used to balance baseline characteristics, yielding 7676 patients in each group. The primary outcome was incident hyperuricemia, defined as serum uric acid >7.0 mg/dL. A secondary outcome was initiation of anti-gout therapy.

**Results:**

During 12 months of follow-up, hyperuricemia occurs more frequently in patients treated with bempedoic acid than in those receiving ezetimibe (hazard ratio [HR] 1.94; 95% confidence interval [CI] 1.58–2.37; p = 0.008). In contrast, initiation of anti-gout therapy does not differ between groups (HR 1.06; 95% CI 0.86–1.29; p = 0.59). Longitudinal changes in lipid parameters, inflammatory markers, and glycemic measures are generally similar between groups.

**Conclusions:**

In real-world clinical practice, bempedoic acid use is associated with a higher incidence of laboratory-defined hyperuricemia compared with ezetimibe, but this increase does not translate into greater use of anti-gout medications. These findings support the continued use of bempedoic acid for additional LDL-C lowering, with awareness of its effect on serum uric acid.

## Plain language summary

High cholesterol is a major cause of heart disease, and for some people current medicines aren’t sufficient to lower their cholesterol. Bempedoic acid is a newer drug that can help reduce certain kinds of cholesterol, but it may raise levels of a compound called uric acid, which can lead to a condition known as gout. We studied over 15,000 patients in U.S. healthcare systems to see whether this medicine increases the risk of hyperuricemia or gout compared with ezetimibe. We found that bempedoic acid caused higher uric acid levels, but most patients did not need gout treatment. This means that while uric acid may increase, serious symptoms are uncommon. These findings provide real-world information on how bempedoic acid affects uric acid levels and the need for gout treatment in clinical practice.

## Abstract (web summary)

Su et al. compare the risk of hyperuricemia in patients treated with bempedoic acid versus ezetimibe using a large real-world dataset from the United States. They find that bempedoic acid increases laboratory hyperuricemia, but this does not result in higher observed use of gout medications.

## Introduction

Hyperlipidemia is a well-recognized and modifiable risk factor for atherosclerotic cardiovascular disease (ASCVD) and remains a significant global public health challenge even with the widespread availability of statins and other lipid-lowering therapies^1,2^. Among lipid parameters, reduction of low-density lipoprotein cholesterol (LDL-C) is the foundational therapeutic target for the prevention of ASCVD, with extensive clinical evidence demonstrating that intensive reduction of LDL-C significantly reduces cardiovascular morbidity and mortality^3,4^. However, many patients do not reach target levels of LDL-C due to inadequate response or statin intolerance, necessitating alternative lipid-lowering strategies^5,6^.

Bempedoic acid, an oral adenosine triphosphate–citrate lyase inhibitor, is approved in the United States, the European Union, and other regions for use in adults with primary hyperlipidemia, including those with or without atherosclerotic cardiovascular disease (ASCVD) or heterozygous familial hypercholesterolemia (HeFH), to lower low-density lipoprotein cholesterol (LDL-C) and reduce cardiovascular risk^7^. It is used primarily in patients with atherosclerotic cardiovascular disease (ASCVD) or heterozygous familial hypercholesterolemia who are unable to tolerate statins or achieve adequate LDL-C reduction with statin therapy alone^8–10^. The CLEAR Harmony, CLEAR Wisdom, and CLEAR Outcomes trials have shown its efficacy in lowering LDL-C and its general safety profile, but these trials also reported small but statistically significant increases in serum uric acid (UA) and a slightly higher incidence of gout, mechanistically attributed to inhibition of renal organic anion transporter 2 (OAT2), which reduces urate clearance^11,12^. Hyperuricemia, defined by elevated serum urate concentrations, is not only a risk factor for gout and nephrolithiasis, but has also been implicated in hypertension, chronic kidney disease, and cardiovascular morbidity^13,14^. Importantly, some lipid-modifying therapies, particularly niacin, have been shown to elevate serum uric acid levels and may precipitate gout in predisposed individuals^15–17^.

Bempedoic acid can increase serum UA by inhibiting organic anion transporter 2 (OAT2) in proximal tubules, thereby reducing urate clearance, whereas ezetimibe does not affect OAT2 activity or urate metabolism^18^. Given the increasing use of bempedoic acid as a statin-sparing agent, especially among older adults with multiple comorbidities, characterizing its metabolic side effect profile is essential for informed clinical decision-making. Although randomized trials have quantified the biochemical impact of bempedoic acid on serum UA, including CLEAR Harmony and CLEAR Outcomes, bempedoic acid was associated with a mean serum UA increase of approximately 0.76 mg/dL and a relatively increased risk of gout compared with placebo (3.1% vs. 2.1%). These findings highlight the potential for urate-related adverse events; however, trial participants were highly selected and closely monitored, limiting the applicability of these results to routine clinical practice. Although bempedoic acid is known to increase serum UA levels, the clinical implications of this effect in real-world settings remain uncertain^19,20^.

To address this gap, we conduct a large-scale retrospective cohort study using the TriNetX U.S. Collaborative Network to evaluate the risk of hyperuricemia associated with bempedoic acid therapy compared to ezetimibe, a non-statin lipid-lowering agent with no known effects on UA metabolism. Our analysis includes more than 15,000 patients with hyperlipidemia matched with the propensity score who initiated bempedoic acid or ezetimibe, allowing us to assess both biochemical results (serum changes in UA) and clinical endpoints (initiation of antigout therapy) within a heterogeneous population in the real world reflective of routine clinical care.

## Methods

### Data sources

The retrospective cohort study used de-identified electronic health record data from the TriNetX Research Network (TriNetX LLC, Cambridge, MA, USA), a federated research platform comprising electronic medical records from approximately 131 million patients across 71 healthcare organizations. Data were accessed on 3 May 2025. The National Cheng Kung University Hospital has institutional access to the TriNetX platform through a data use agreement with TriNetX LLC. All data available through TriNetX are de-identified in accordance with the Health Insurance Portability and Accountability Act (HIPAA) Privacy Rule.

The use of de-identified TriNetX data was reviewed by the Institutional Review Board of National Cheng Kung University Hospital and determined to be exempt from full review (Approval No. B-ER-114-232). The TriNetX platform itself operates under a waiver of informed consent granted by the Western Institutional Review Board (WIRB), as all data are de-identified. The study was carried out in accordance with the Strengthening the Reporting of Observational Studies in Epidemiology (STROBE) guidelines.

### Study design

This retrospective cohort study was structured to resemble a randomized comparison, with clearly defined index, baseline, and follow-up periods. The index date was defined as the first recorded prescription for study medications. Baseline characteristics were assessed using data prior to the index date. Adult patients (aged ≥ 18 years) with a diagnosis of hyperlipidemia who initiated bempedoic acid or ezetimibe were eligible. To isolate incident cases of hyperuricemia, individuals with a prior diagnosis of hyperuricemia or the use of anti-gout therapy before the index date were excluded.

Eligible patients were classified into two exposure cohorts: (i) new users of bempedoic acid and (ii) new users of ezetimibe. Ezetimibe was selected as a comparator due to its common use in patients who are statin-intolerant and the lower risk of increased UA level, providing a clinically appropriate control to evaluate the independent effects of bempedoic acid. We specified a hypothetical trial protocol to shape the study design and emulate the components of a trial drawing on observational data (Supplementary Figs. 1 and 2, Supplementary Tables 1 and 2).

The study period extended from January 1, 2020, to May 3, 2025, corresponding to the period of bempedoic acid availability in the TriNetX U.S. Collaborative Network. To identify new users, patients were required to have no prescription record for the index medication during the year prior to the index date (washout period). A 12-month look-back period before the index date was applied to assess baseline comorbidities, concomitant medications, and other covariates. Follow-up continued for post-index or until death, treatment discontinuation, or the end of data availability.

### Outcomes of interest

The primary outcome of interest was the incidence of hyperuricemia, defined as a UA level > 7.0 mg/dL, consistent with the cutoff used in pivotal bempedoic acid trials (CLEAR Harmony, CLEAR Wisdom, and CLEAR outcomes). This threshold corresponds closely to the point of urate supersaturation in plasma. Although the 2020 American College of Rheumatology (ACR) guidelines^21^ define hyperuricemia as > 6.8 mg/dL, the theoretical limit of solubility of urate under physiological conditions, most large-scale epidemiological and interventional studies have continued to adopt 7.0 mg/dL for clinical relevance and comparability across datasets. Alternative definitions in the literature have proposed lower thresholds (e.g., > 6.8 mg/dL, the theoretical saturation point of urate under physiological conditions), particularly in populations with high cardiovascular risk; however, the 7.0 mg/dL threshold remains the most widely applied in large-scale epidemiological and clinical studies. Because the diagnostic coding for gout is inconsistently available in TriNetX, the initiation of anti-gout medications (e.g., allopurinol, febuxostat, and colchicine) was used as a validated proxy for incident gout events. Two complementary definitions were applied. Biochemical hyperuricemia was defined as serum UA > 7.0 mg/dL, while clinically significant hyperuricemia required both elevated UA and the initiation of anti-gout therapy during follow-up. Patients with elevated UA but no anti-gout therapy were categorized as having asymptomatic hyperuricemia, whereas those who initiated anti-gout therapy without an elevated UA were considered discordant and analyzed separately. Anti-gout medications included xanthine oxidase inhibitors (allopurinol, febuxostat), and colchicine. Secondary outcomes included changes in serum UA levels, CRP, LDL-C, high-density lipoprotein cholesterol (HDL-C), triglycerides, and hemoglobin A1c (HbA1c). These biomarkers were assessed at baseline and 3, 6, and 12 months after the index date.

### Covariates

To control for possible confounding, the analysis was adjusted for a range of demographic and clinical variables. This included age, sex, race, and the presence of type 1 or type 2 diabetes mellitus. Cardiovascular comorbidities were also included, including coronary artery disease, hypertension, ischemic heart disease, ischemic stroke, peripheral artery disease, non-traumatic intracerebral hemorrhage, chronic kidney disease, end-stage renal disease, and heart failure. Laboratory covariates included baseline lipid parameters (total cholesterol, LDL-C, HDL-C, and triglycerides) and inflammatory markers (CRP). Uric acid levels were also incorporated; although UA is not a classical inflammatory marker, it has been associated with cardiovascular risk and metabolic disturbances. All comorbid conditions were identified using the relevant ICD-10-CM diagnostic codes available in the TriNetX database.

### Statistical analysis

Continuous variables were summarized as means with standard deviations (mean ± SD), and categorical variables were expressed as absolute counts and percentages. To ensure baseline comparability between treatment groups, a propensity score matching was performed prior to the primary, subgroup, and sensitivity analyses. Propensity scores were estimated using multivariable logistic regression that included age, sex, race, baseline comorbidities (e.g., hypertension, diabetes mellitus, chronic kidney disease, coronary artery disease, heart failure), and concurrent cardiovascular medications. Balance between matched cohorts was assessed using the absolute standardized mean difference (SMD), with an SMD < 0.1 indicating adequate balance. After matching, no further covariate adjustment was applied in the primary analysis, as covariate balance had been achieved.

The primary analysis estimated the risk of hyperuricemia incident among patients receiving bempedoic acid versus ezetimibe using Cox proportional hazards regression, with results expressed as Hazard ratios (HRs) and 95% CIs. To evaluate the consistency of our findings, we conducted prespecified subgroup analyses across clinically relevant categories that could potentially modify the relationship between bempedoic acid use and hyperuricemia. Subgroups included age (< 65 vs. ≥ 65 years), sex, baseline UA status (normal vs. elevated), presence of diabetes mellitus, hypertension, chronic kidney disease, and baseline use of diuretics. These factors were selected based on their known associations with UA metabolism and gout risk. For each subgroup, HRs and 95% CIs for the outcomes of interest, incident hyperuricemia and initiation of anti-gout therapy, were re-estimated using Cox proportional hazards models within the propensity score-matched cohort. In the primary analysis, exposure groups were defined by the index medication initiated; patients receiving the fixed-dose bempedoic acid/ezetimibe combination were not excluded. A sensitivity analysis was additionally performed excluding all patients with any record of fixed-dose combination therapy.

Secondary biochemical outcomes, including changes in LDL-C, HDL-C, triglycerides, total cholesterol, CRP, HbA1c, and UA were evaluated at 3, 6, and 12 months. Patients were included in each time point-specific analysis only if the corresponding laboratory values were available. Given the variability in the frequency of tests and the missing data between sites, these results were summarized descriptively rather than compared statistically between treatment groups. This approach minimized potential bias related to non-random laboratory measurement. For secondary laboratory outcomes, the TriNetX platform provides aggregated summary statistics. The reported numbers of patients at each follow-up time point reflect the total number of patients with at least one laboratory record available within that time window and do not represent analyte-specific sample sizes. All analyses were performed within the TriNetX analytics platform and were prespecified based on clinically relevant hypotheses and prior evidence.

### Ethics approval

The study protocol received approval from National Cheng Kung Universiy Hospital’s Institutional Review Board (B-ER-114-232).

## Results

### Selection and matching of cohorts

**Fig. 1:**
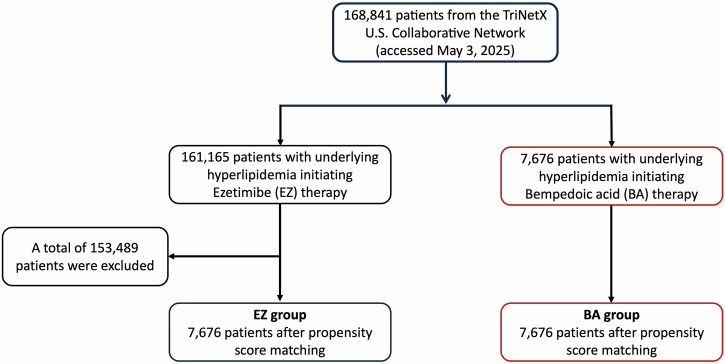
Flow diagram of patient selection and cohort matching. Flow diagram of cohort selection for bempedoic acid versus ezetimibe in the TriNetX U.S. Collaborative Network.

Figure 1 illustrates the cohort selection process and shows the number of patients included and excluded to derive the final propensity score-matched cohorts. We initially identified 168,841 adult patients with underlying hyperlipidemia from the TriNetX U.S. Collaborative Network, accessed on 3 May 2025. Among these, 7676 patients had started bempedoic acid therapy, while 161,165 patients had initiated ezetimibe therapy during the study period. To ensure comparable baseline characteristics between treatment groups, we applied 1:1 propensity score matching using variables that included age, sex, race, comorbidities, and relevant laboratory values. Following the application of the matching criteria, 153,489 patients from the ezetimibe cohort were excluded, resulting in 7676 patients in the ezetimibe group who were successfully matched with 7676 patients in the bempedoic acid group. This matching strategy provided two well-balanced cohorts for subsequent comparative analyzes of the incidence of hyperuricemia, changes in serum UA levels, and related clinical outcomes, including the initiation of anti-gout therapy. This careful selection and matching process facilitated a robust evaluation of the real-world impact of bempedoic acid on UA metabolism and associated clinical endpoints relative to a non-statin lipid-lowering comparator in a large, diverse patient population.

### Baseline characteristics

The final cohorts matched demonstrated comparable demographic and clinical profiles, as summarized in Supplementary Table 3. A total of 168,841 patients were initially identified, comprising 7676 patients in the bempedoic acid group and 161,165 patients in the ezetimibe group. Before the matching of the propensity score, there were notable differences in baseline characteristics between the groups. The patients in the bempedoic acid group were slightly older (mean age: 65.60 ± 10.60 years vs. 64.30 ± 11.10 years; SMD = 0.120) and were more likely to be female (65.80% vs. 58.90%), while a higher proportion of the patients in the ezetimibe group were male (41.10% vs. 34.20%; SMD = 0.142). There were also slight variations in the prevalence of comorbidities, including type 2 diabetes mellitus (31.80% vs 26.80%; SMD = 0.110), heart failure (11.40% vs. 8.50%; SMD = 0.098) and cerebrovascular disease.

In addition to baseline demographics and comorbidities, the concomitant use of lipid-modifying and UA-influencing agents was evaluated. Before matching, the use of statins (54.77% vs. 37.34%), evolocumab (2.48% vs. 11.18%), and alirocumab (0.92% vs. 4.67%) differed significantly between ezetimibe and bempedoic acid users. Following propensity score matching, the distributions of these agents were well balanced (all SMDs < 0.1). Post-matching, the frequencies of medication use were as follows: statins 37.34% vs. 37.97%, fibrates 2.91% vs. 3.39%, evolocumab 11.18% vs. 11.01%, alirocumab 4.67% vs. 4.22%, niacin 0.55% vs. 0.59%, diuretics 23.67% vs. 22.27%, SGLT2 inhibitors 5.54% vs. 5.64%, and losartan 13.01% vs. 12.57% for bempedoic acid and ezetimibe, respectively.

Laboratory parameters demonstrated significant baseline differences between the groups before matching. The bempedoic acid cohort had higher mean levels of total cholesterol (229.80 ± 49.90 mg / dL vs 219.40 ± 48.80 mg/dL; SMD = 0.21), LDL-C (146.70 ± 43.10 mg / dL vs 137.90 ± 41.30 mg/dL; SMD = 0.21), and HDL-C (49.90 ± 20.20 mg / dL vs 47.80 ± 19.50 mg/dL; SMD = 0.11). UA levels were also lower in the bempedoic acid group (5.40 ± 1.20 mg / dL vs 5.70 ± 2.00 mg/dL; SMD = 0.19), while CRP and HbA1c values were higher in the ezetimibe group. These results indicate successful control of confounders and comparability of treatment cohorts.

### Duration of follow-up

The study population was followed for a period of 12 months. All outcomes were evaluated at multiple intervals throughout this duration of follow-up using matched cohorts derived from adjustment of the propensity score.

#### Primary outcome: Incidence of hyperuricemia and Initiation of anti-gout therapy

As shown in Table 1, in the propensity score–matched cohorts revealed a significantly higher incidence of hyperuricemia in patients treated with bempedoic acid compared to those receiving ezetimibe. During the 12-month follow-up period, 266 patients (3.50%) in the bempedoic acid group developed hyperuricemia compared to 149 patients (1.90%) in the ezetimibe group. The difference between the two treatment arms was statistically significant, as confirmed by the log-rank test (χ² = 43.16, df = 1, *p* < 0.01). Cox proportional hazards modeling demonstrated that bempedoic acid use was associated with a markedly increased risk of developing hyperuricemia: HR: 1.94; 95% CI: 1.58−2.37; *p* = 0.008.

**Table. 1:** Comparative incidence of hyperuricemia and the initiation of antigout therapy

Outcome	Treatment Group	Patients (*n*)	Events (n)	Hazard Ratio (95% CI)	*p*-value
Hyperuricemia	BA	7,675	266	1.94 (1.58−2.37)	*p* = 0.008
	EZ	7,675	149		
Initiation of Treatment for Hyperuricemia	BA	7,675	187	1.06 (0.86−1.29)	*p* = 0.59
	EZ	7,675	190		

*BA* bempedoic acid, *EZ* ezetimibe.

Although this was a high risk, the initiation of anti-gout therapy did not differ significantly between the groups. A total of 187 patients (2.40%) in the bempedoic acid cohort and 190 patients (2.50%) in the ezetimibe group-initiated treatment with anti-gout medications. The log-rank test produced a non-significant result (χ² = 0.28, df = 1, *p* = 0.59). Similarly, the adjusted risk ratio for the initiation of anti-gout therapy in the bempedoic acid group was HR: 1.06; 95% CI: 0.86−1.29; *p* = 0.59. These findings suggest that although bempedoic acid is associated with a higher incidence of hyperuricemia, this does not translate into a significantly increased need for pharmacological treatment of the condition.

In addition to the primary outcome analyses, discordant outcome patterns were examined. Among patients who developed biochemical hyperuricemia (serum uric acid > 7.0 mg/dL) without subsequent initiation of anti-gout therapy, 185 patients (2.41%) were in the bempedoic acid group, and 86 patients (1.12%) were in the ezetimibe group. Conversely, among patients who initiated anti-gout therapy without a documented elevation in serum uric acid, 134 patients (1.75%) were in the bempedoic acid group, and 121 patients (1.58%) were in the ezetimibe group. These discordant cases were analyzed separately and did not materially alter the primary findings.

In a sensitivity analysis excluding patients who received the fixed-dose bempedoic acid/ezetimibe combination tablet, the association between bempedoic acid use and incident hyperuricemia remained significant(HR: 2.35; 95% CI: 1.61–3.45; *p* < 0.01), and no difference was observed in the initiation of anti-gout therapy between groups (HR: 1.13; 95% CI: 0.81–1.58; *p* = 0.39).

#### Secondary outcomes

**Fig. 2:**
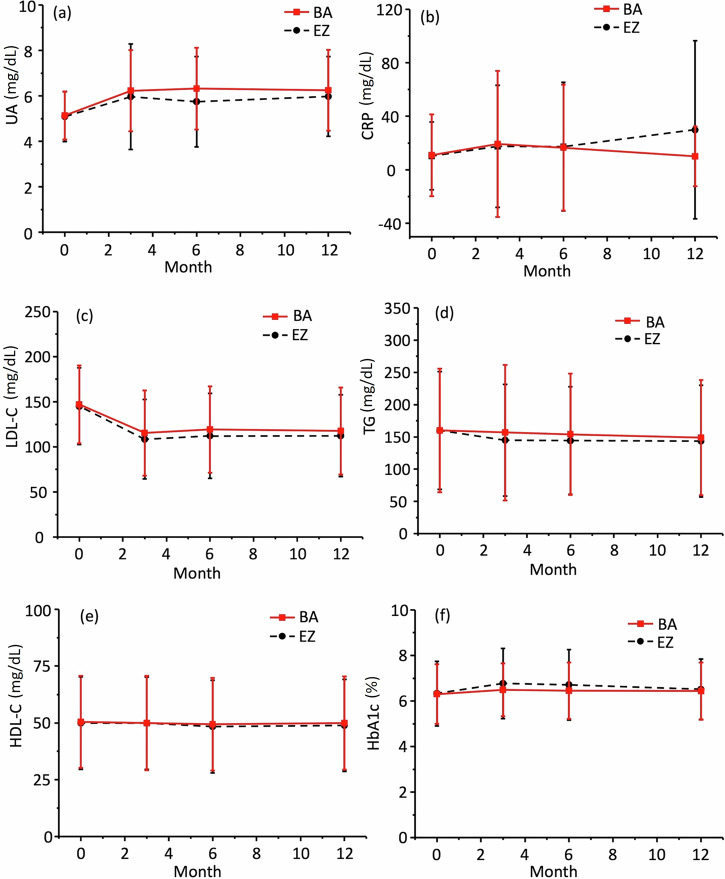
Longitudinal changes in metabolic and inflammatory biomarkers: bempedoic acid versus ezetimibe. Metabolic and inflammatory biomarkers were followed for 12 months in patients treated with bempedoic acid (red lines) or ezetimibe (black dashed lines). Laboratory results are presented as mean ± standard deviation (SD). **a** UA; **b** CRP; **c** LDL-C; **d** TG; **e** HDL-C; **f** HbA1c.

Serial laboratory assessments were carried out to evaluate the longitudinal effects of bempedoic acid (BA) and ezetimibe (EZ) on key metabolic and inflammatory biomarkers over a 12-month period. Figure 2 illustrates the progression over time of serum UA, CRP, LDL-C, TG, HDL-C, and HbA1c at baseline, 3, 6, and 12 months in patients treated with BA and EZ.

Figure 2 presents the longitudinal trends in serum UA, CRP, LDL-C, triglycerides, HDL-C, and HbA1c levels over 12 months of follow-up in the bempedoic acid and ezetimibe cohorts. Corresponding mean values and standard deviations for each time point are summarized in Table 3.

During follow-up, both bempedoic acid and ezetimibe showed generally similar effects on overall lipid and metabolic parameters overall; however, differences were observed in specific biomarkers, particularly serum UA and C-reactive protein. Patients treated with bempedoic acid exhibited a more pronounced increase in serum UA levels, increasing from a baseline of 5.13 ± 1.05 mg/dL to 6.24 ± 1.78 mg/dL in 12 months, compared to an increase of 5.09 ± 1.10 mg/dL to 5.97 ± 1.75 mg/dL in the ezetimibe group. Although both therapies led to reductions in LDL-C and total cholesterol over the 12-month follow-up period, the overall patterns of lipid lowering were similar between the bempedoic acid and ezetimibe cohorts. CRP levels, a surrogate marker of systemic inflammation, fluctuated modestly over time in both treatment groups, without a consistent pattern of difference between bempedoic acid and ezetimibe (Supplementary Table 4). Triglyceride and HDL-C levels remained relatively stable between both treatment groups. The glucose control, measured by HbA1c, remained stable throughout the study period in both cohorts. These findings, summarized in Supplementary Table 5, underscore the metabolic stability and lipid-lowering efficacy of both agents, while highlighting the higher elevation of UA associated with bempedoic acid therapy.

Findings from subgroups were consistent with the primary results, showing a modestly higher risk of hyperuricemia associated with bempedoic acid use across most of the subgroups. This association was generally observed in most subgroups, with the exception of patients who smoke (HR 1.50; 95% CI 0.79–2.84), those without obesity (HR 1.28; 95% CI 0.74–2.21), and individuals without hypertension (HR 2.10; 95% CI 0.90–4.90), where confidence intervals included null. Importantly, in all subgroups examined, the use of bempedoic acid was not associated with an increased need for the treatment of hyperuricemia. These findings were further supported by a series of sensitivity analyses, which underscoring the robustness of our results.

## Discussion

In this comprehensive retrospective cohort study using data from the TriNetX U.S. Collaborative Network, we examined the incidence of hyperuricemia in patients with hyperlipidemia treated with bempedoic acid or ezetimibe. After a precise match of the propensity score to 1: 1, we found that the initiation of bempedoic acid was significantly associated with a higher incidence of hyperuricemia compared to ezetimibe, with a HR of 1.94 (95% CI: 1.58–2.37; p = 0.008). Even with this biochemical finding, there was no corresponding increase in the initiation of antigout therapy between treatment groups (HR: 1.06; 95% CI: 0.86–1.29; *p* = 0.59), indicating that the observed hyperuricemia was predominantly asymptomatic and clinically mild.

Our real-world data support the findings of the clinical trials, including the CLEAR Harmony, CLEAR Wisdom, and the more recent CLEAR Outcomes trial^7–10^. In the CLEAR Outcomes trial, bempedoic acid was associated with modest increases in serum UA and a slightly higher incidence of gout (3.1% vs. 2.1%), consistent with the mechanism of inhibition of renal OAT2. When compared with the CLEAR Outcomes trial, our real-world analysis demonstrated a similar relative increase in hyperuricemia, but no corresponding increase in gout treatment initiation. This discrepancy is likely explained by the different definitions of outcomes. That is, we defined hyperuricemia using recorded serum uric acid values and medication records, whereas the CLEAR study used investigator-reported adverse events. It is also plausible that patients with mild or self-limited gout flares managed with non-specific anti-inflammatory or analgesic agents were not captured in our dataset, resulting in an underestimation of symptomatic gout. Our findings complement those of the CLEAR Outcomes program and its recent sub-analysis by Ray et al., who reported that the concomitant use of urate-lowering therapies may attenuate the risk of gout in patients receiving bempedoic acid^22^. This reinforces the clinical importance of monitoring UA levels and considering preventive management in high-risk individuals. Furthermore, our median follow-up duration was shorter than that of CLEAR results (approximately 12 vs. 40 months), which may have limited statistical power to detect small differences in incident gout. Therefore, the absence of an association between bempedoic acid use and gout should be interpreted cautiously, as a Type II error cannot be excluded. Conversely, multiple secondary comparisons may increase the potential for Type I error, further emphasizing the need for replication in longer-term prospective studies. However, it is important to emphasize that participants in CLEAR trials represented a highly selected population: they were routinely monitored, underwent regular laboratory tests, and generally had higher adherence and more structured clinical follow-up.

In contrast, our study reflects the complexities of routine clinical practice, where patients are more heterogeneous, and monitoring is often less frequent or structured. This discrepancy can lead to differences in the clinical manifestation and detection of complications related to hyperuricemia. In real-world settings, underlying inflammatory conditions or renal dysfunction can go undetected for longer periods, potentially amplifying the biological impact of UA accumulation. On the contrary, the absence of intensive surveillance might underrecognize mild or transient cases, as evidenced by the relatively low rate of anti-gout medication initiation in our cohort. Our findings are consistent with recent pharmacovigilance data from the FDA Adverse Event Reporting System (FAERS), which also identified hyperuricemia and gout among the most frequently reported metabolic adverse events associated with bempedoic acid use, though the overall incidence remained low^23^.

This contextual difference underscores the rationale for conducting our study: to determine whether hyperuricemia observed in highly controlled trial environments translates into meaningful clinical consequences in a broader population. Our findings demonstrate that while bempedoic acid leads to a measurable increase in serum UA, this elevation rarely translates into clinically significant outcomes that require treatment.

Furthermore, serial evaluations of metabolic and inflammatory biomarkers over 12 months demonstrated that both bempedoic acid and ezetimibe were associated with comparable reductions in LDL-C and total cholesterol over the 12-month follow-up period. CRP level changes were variable and did not demonstrate a consistent difference between bempedoic acid and ezetimibe in this real-world analysis, although in prior randomized clinical trials, bempedoic acid has been shown to reduce median high-sensitivity C-reactive protein levels by ~20–30%, suggesting potential anti-inflammatory effects beyond LDL-C lowering ^7–10^.

Given its favorable lipid-lowering profile, metabolic neutrality, and manageable safety concerns, bempedoic acid represents a valuable non-statin oral therapeutic option, particularly for patients who are statin intolerant. The elevated risk of hyperuricemia should not preclude its use but should be contextualized within its overall benefit-risk profile. Selective monitoring may be appropriate in high-risk individuals, but the absence of widespread clinical consequences supports continued use without routine UA surveillance, according to current lipid management guidelines^3^. Our real-world data support the findings of the clinical trial, showing that bempedoic acid increases serum UA levels, but clinically significant hyperuricemia remains low. Clinicians must distinguish between biochemical and clinical hyperuricemia when evaluating bempedoic acid therapy, especially in patients with complex comorbidities.

This study has several notable key strengths. In particular, the use of the TriNetX U.S. federated research network provided access to a large, demographically, and geographically diverse patient cohort drawn from more than 100 healthcare organizations. This diversity improves the external validity and generalizability of our findings in real-world clinical settings. Second, the adoption of a target trial emulation framework allowed us to structure the observational analysis in a manner that approximates the rigor and design principles of a randomized controlled trial, thus reducing bias related to treatment assignments. Third, we employ robust propensity score matching to balance key baseline characteristics, including cardiovascular comorbidities, lipid parameters, and initial serum UA concentrations. This methodological approach strengthened internal validity by minimizing confounding and ensuring comparability between treatment groups. Fourth, because TriNetX aggregates laboratory data as means and standard deviations, we were unable to report CRP as medians with interquartile ranges, which would be more appropriate for non-normally distributed biomarkers. Fifth, given the observational design and limited follow-up duration, the potential for both Type I and Type II statistical errors exists. However, the observed association is supported by well-established biological mechanisms: bempedoic acid increases serum UA by inhibiting renal OAT transporters, and the direction of effect is consistent with prior clinical evidence, including findings from CLEAR outcomes. Sixth, since laboratory assessments in the TriNetX network are performed according to clinical indication rather than protocol-driven schedules, not all laboratory parameters were available for every patient or at every time point. This reflects the inherent nature of real-world data, which captures clinical practice variability but may lead to incomplete laboratory reporting. We acknowledge this limitation and have presented laboratory results as aggregated summary statistics, consistent with the structure of the database.

However, several limitations must be recognized. As an observational study using retrospective electronic health record (EHR) data, the possibility of residual confounding cannot be completely excluded, despite the use of advanced statistical adjustment methods. In addition, initiation of anti-gout therapy was used as a proxy for clinically significant hyperuricemia and should not be interpreted as a definitive diagnosis of gout. Patients may have experienced gout without receiving pharmacologic treatment, may have received treatment outside of the captured healthcare network, or may have been prescribed anti-gout medications for indications other than acute gout. In addition, gout events were not directly adjudicated or analyzed in this study, potentially leading to an underestimation of clinically symptomatic events. Furthermore, laboratory measurements such as serum UA levels were not collected uniformly between institutions and may vary in frequency and timing, introducing potential measurement bias. A notable limitation of this study is the substantial variability in CRP levels observed in the cohort, which may reflect differences in data collection methods between institutions contributing to the TriNetX database. This variability can affect the reliability of CRP as a biomarker in our analysis. CRP was reported as mean ± standard deviation rather than median (interquartile range), as the TriNetX analytics platform does not consistently provide distributional metrics beyond summary statistics for laboratory variables at each time point. This constraint is consistent with prior analyses of TriNetX laboratory data reporting^24^. Lastly, medication adherence was inferred from prescription records rather than direct observation, which may not accurately reflect true patient behavior and could influence the interpretation of the outcome.

## Conclusions

In conclusion, the present large real-world cohort study demonstrates that while bempedoic acid therapy is significantly associated with an increased incidence of laboratory-defined hyperuricemia compared to ezetimibe, this biochemical elevation does not translate into a clinically significant increase in the use of anti-gout medications. The observed hyperuricemia appears to be mild, predominantly asymptomatic, and self-limited, suggesting limited clinical consequences in routine practice. These findings support the general safety profile of bempedoic acid as an effective non-statin lipid-lowering agent, particularly for statin-intolerant patients. Although clinicians should be aware of this possible metabolic side effect, routine UA surveillance or intervention may not be necessary for most patients, except those with known risk factors for gout or renal failure.

## Supplementary information

Supplementary Info

Transparent Peer Review file

## Data Availability

The data that support the findings of this study are available from the TriNetX Research Network (TriNetX LLC), but restrictions apply to the availability of these data, which were used under license for the current study and are not publicly available. Researchers may obtain access to the TriNetX platform through institutional subscription and approval by TriNetX.

## References

[1] Nelson, R. H. Hyperlipidemia as a Risk Factor for Cardiovascular Disease. *Prim. Care: Clin. Off. Pract.* **40**, 195–211 (2013). 10.1016/j.pop.2012.11.003 PMC3572442 23402469

[2] Rosenblit, P. D. Extreme Atherosclerotic Cardiovascular Disease (ASCVD) Risk Recognition. *Curr. Diab Rep.* **19**, 61 (2019). 31332544 10.1007/s11892-019-1178-6

[3] Wilson, P. W. F. et al. Systematic Review for the 2018 AHA/ACC/AACVPR/AAPA/ABC/ACPM/ADA/AGS/APhA/ASPC/NLA/PCNA Guideline on the Management of Blood Cholesterol: A Report of the American College of Cardiology/American Heart Association Task Force on Clinical Practice Guidelines. *Circulation* **139**, (2019). 10.1161/CIR.0000000000000626 30586775

[4] Liu, P. Y. Reappraisal of the New Clinical Pathway National Consensus on Lipid Profile in Taiwan 2025: Where Do We Stand Now? *Acta Cardiol Sin.* **41**, 166–168 (2025). 40123606 10.6515/ACS.202503_41(2).20250210A PMC11923779

[5] Stroes, E. S. et al. Statin-associated muscle symptoms: impact on statin therapy—European Atherosclerosis Society Consensus Panel Statement on Assessment, Aetiology and Management. *Eur. Heart J.* **36**, 1012–1022 (2015). 25694464 10.1093/eurheartj/ehv043 PMC4416140

[6] Di Minno, A. et al. Efficacy and Safety of Bempedoic Acid in Patients With Hypercholesterolemia: Systematic Review and Meta-Analysis of Randomized Controlled Trials. *J. Am.**Heart Assoc*. **9**, e016262 (2020). 10.1161/JAHA.119.016262 PMC7792250 32689862

[7] Mach, F. et al. ESC/EAS Scientific Document Group. 2025 Focused Update of the 2019 ESC/EAS Guidelines for the management of dyslipidaemias. *Eur. Heart J.* **46**, 4359–4378 (2025). 40878289 10.1093/eurheartj/ehaf190

[8] Ray, K. K. et al. Safety and Efficacy of Bempedoic Acid to Reduce LDL Cholesterol. *N. Engl. J. Med.* **380**, 1022–1032 (2019). 30865796 10.1056/NEJMoa1803917

[9] Yarrarapu, S. N. S. et al. Comprehensive review of statin-intolerance and the practical application of Bempedoic Acid. *J. Cardiol.* **84**, 22–29 (2024). 38521120 10.1016/j.jjcc.2024.03.006

[10] Nissen, S. E. et al. Bempedoic Acid and Cardiovascular Outcomes in Statin-Intolerant Patients. *N. Engl. J. Med.* **388**, 1353–1364 (2023). 36876740 10.1056/NEJMoa2215024

[11] Laufs, U. et al. Efficacy and Safety of Bempedoic Acid in Patients With Hypercholesterolemia and Statin Intolerance. *J. Am. Heart Assoc.* **8**, e011662 (2019). 10.1161/JAHA.118.011662 PMC6509724 30922146

[12] Biolo, G. et al. Mechanism of action and therapeutic use of bempedoic acid in atherosclerosis and metabolic syndrome. *Front. Cardiovasc. Med.* **9**, 1028355 (2022). 10.3389/fcvm.2022.1028355 PMC9650075 36386319

[13] Richette, P. et al. 2016 updated EULAR evidence-based recommendations for the management of gout. *Ann. Rheum. Dis.* **76**, 29–42 (2017). 27457514 10.1136/annrheumdis-2016-209707

[14] Zhang, S. et al. Hyperuricemia and Cardiovascular Disease. *Curr. Pharm. Des.* **25**, 700–709 (2019). 30961478 10.2174/1381612825666190408122557

[15] D’Agostino, R. B., Kannel, W. B., Stepanians, M. N. & D’Agostino, L. C. A comparison between lovastatin and gemfibrozil in the treatment of primary hypercholesterolemia. *Am. J. Cardiol.* **69**, 28–34 (1992). 1729864 10.1016/0002-9149(92)90671-k

[16] Davidson, M. H. & Toth, P. P. Comparative effects of lipid-lowering therapies. *Prog. Cardiovasc Dis.* **47**, 73–104 (2004). 15586350 10.1016/j.pcad.2004.04.007

[17] Leung, N., Yip, K., Pillinger, M. H. & Toprover, M. Lowering and Raising Serum Urate Levels: Off-Label Effects of Commonly Used Medications. *Mayo Clin. Proc.* **97**, 1345–1362 (2022). 35787862 10.1016/j.mayocp.2022.02.027 PMC9575594

[18] Ballantyne, C. M. et al. Role of Bempedoic Acid in Clinical Practice. *Cardiovasc Drugs Ther.* **35**, 853–864 (2021). 33818688 10.1007/s10557-021-07147-5 PMC8266788

[19] De Filippo, O. et al. Safety and efficacy of bempedoic acid: a systematic review and meta-analysis of randomised controlled trials. *Cardiovasc Diabetol.* **22**, 324 (2023). 38017541 10.1186/s12933-023-02022-z PMC10685600

[20] Khan, M. U., Khan, M. Z., Munir, M. B., Balla, S. & Khan, S. U. Meta-analysis of the Safety and Efficacy of Bempedoic Acid. *Am. J. Cardiol.* **131**, 130–132 (2020). 32711805 10.1016/j.amjcard.2020.06.028

[21] FitzGerald, J. D. et al. 2020 American College of Rheumatology Guideline for the Management of Gout. *Arthritis Care Res (Hoboken)* **72**, 744–760 (2020). 32391934 10.1002/acr.24180 PMC10563586

[22] Ray, K. K. et al. Association of Uric Acid-Lowering Therapies on Gout Frequency with Bempedoic Acid. *Clin. Insights CLEAR Outcomes JACC Adv.* **4**, 102207 (2025). 10.1016/j.jacadv.2025.102207 PMC12510181 41014810

[23] Wang, X. et al. Novel insights into post-marketing AEs associated with bempedoic acid: a comprehensive analysis utilizing the FAERS database. *Expert Opin. Drug Saf*. 1–8. 10.1080/14740338.2025.2468868. Online ahead of print. (2025). 10.1080/14740338.2025.2468868 39960262

[24] Wang, J., Tsai, K. W., Lu, C. L. & Lu, K. C. Analyzing clinical laboratory data outcomes in retrospective cohort studies using TriNetX. *Biochem. Med. (Zagreb)* **35**, 030502 (2025). 40822843 10.11613/BM.2025.030502 PMC12334939

