# Supplementary material for: Hyperuricemia risk in bempedoic acid-treated hyperlipidemic patients
Source: Commun Med (Lond). 2026 Apr 1;6:328. doi: 10.1038/s43856-026-01545-2 (PMC13243465; doi:10.1038/s43856-026-01545-2)
Supplement: Supplementary file 1 — Supplementary Info [file 43856_2026_1545_MOESM1_ESM.pdf]

Supplementary Table 1. Specification and emulation of a target trial

Component	Target trial	Emulated trial using real-world data
Aim	To evaluate the relative effect of Bempedoic acid versus Ezetimide on hazards of hyperuriecemia and anti-gout therapy in patients with hyperlipidemia.	Same
Eligibility	Adult patients aged ≥18 years diagnosed with hyperlipidemia without previously diagnosed hyperuricemia or anti-gout therapy<br>No prior use of target medications	Same
Treatment strategies	1. Receiving Bempedoic acid treatment<br>2. Receiving Ezetimide treatment	Same
Treatment assignment	Eligible patients are randomly assigned to the treatment groups (the same probability of treatment assignment between the treatment groups)	Using propensity score matching to generate a study population with similar probability of treatment assignment between groups.
Follow-up	Follow-up begins at treatment assignment and ends at occurrence of outcomes	Same (the assignment and initiation of the treatment occur at the same time in the real-world scenario)
Outcome	Hyperuricemia(>7mg/dL), anti-gout therapy (allopurinol, colchicine, febuxostat)	Same
Statistical analysis	Cox proportional hazards model	Same

Supplementary Table 2. Diagnosis and procedure codes for study outcome and comorbidities

Diseases	ICD-10-CM codes
**Study outcome**	
Hyperuricemia	E79
Anti-gout therapy	
Allopurinol	NLM:RXNORM:519
Colchicine	NLM:RXNORM:2683
Febuxostat	NLM:RXNORM:73689
**Inclusion criteria**	
Hyperlipidemia	E78.5
Use of Bempedoic acid	NLM:RXNORM:2282403
Use of Ezetimide	NLM:RXNORM:341248
**Exclusion criteria**	
Previous Hyperuricemia	E79
Previous Anti-gout therapy	
Allopurinol	NLM:RXNORM:519
Colchicine	NLM:RXNORM:2683
Febuxostat	NLM:RXNORM:73689
**Covariates**	
Cardiovascular comorbidities	
Type 1 diabetes mellitus	E10
Type 2 diabetes mellitus	E11
Hypertensive diseases	I10-I1A
Ischemic heart diseases	I20-I25
Cerebral infarction	I63
Nontraumatic intracerebral hemorrhage	I61
Chronic kidney disease, stage 3 (moderate)	N18.3
End stage renal disease	N18.6
Heart failure	I50

Occlusion and stenosis of precerebral arteries, not resulting in cerebral infarction	I65
Laboratory data	
Cholesterol in LDL [Mass/volume] in Serum or Plasma	9002
C reactive protein [Mass/volume] in Serum, Plasma or Blood	9063
Hemoglobin A1c/Hemoglobin.total in Blood	9037
Cholesterol [Mass/volume] in Serum or Plasma	9000
Cholesterol in HDL [Mass/volume] in Serum or Plasma	9001
Triglyceride [Mass/volume] in Serum, Plasma or Blood	9004
Urate [Mass/volume] in Serum or Plasma	9071

Supplementary Table 3. Baseline characteristics of the study cohorts before and after the matching of the propensity score.

**Characteristics**	**No. of patients in original cohort (%)**			**No. of patients after propensity score matching (%)**		
	**BA n = 7,676**	**EZ N=161,165**	**SMD**	**BA n = 7,676**	**EZ N = 7,676**	**SMD**
Age(yr), Median (IQR)	65.60 +/- 10.60	64.30 +/- 11.10	0.12	65.60 +/- 10.60	65.30 +/- 10.60	0.02
White	5487 (71.50%)	115913 (71.90%)	0.01	5487 (71.50%)	5492 (71.60%)	<0.01
Male	2624 (34.20%)	66176 (41.10%)	0.14	2624 (34.20%)	2667 (34.70%)	0.01
Asian	414 (5.40%)	5664 (3.50%)	0.09	413 (5.40%)	423 (5.50%)	<0.01
Type 1 diabetes mellitus	144 (1.90%)	3342 (2.10%)	0.01	143 (1.90%)	123 (1.60%)	0.02
Type 2 diabetes mellitus	2060 (26.80%)	51310 (31.80%)	0.11	2059 (26.80%)	1966 (25.60%)	0.03
Hypertensive disease	4987 (65.00%)	108793 (67.50%)	0.05	4986 (65.00%)	4963 (64.70%)	<0.01
Ischemia heart disease	2764 (36.00%)	56223 (34.90%)	0.02	2763 (36.00%)	2729 (35.60%)	<0.01
Cerebral infarction	290 (3.80%)	10340 (6.40%)	0.12	290 (3.80%)	278 (3.60%)	<0.01
Nontraumatic intracranial hemorrhage	25 (0.30%)	827 (0.50%)	0.03	25 (0.30%)	32 (0.40%)	0.01
Chronic kidney disease, stage 3 (Moderate)	564 (7.30%)	12986 (8.10%)	0.03	563 (7.30%)	513 (6.70%)	0.03

End stage renal disease	27 (0.40%)	1482 (0.90%)	0.07	27 (0.40%)	32 (0.40%)	0.01
Heart failure	652 (8.50%)	18405 (11.40%)	0.10	652 (8.50%)	610 (7.90%)	0.02
Occlusion and stenosis of precerebral arteries, not resulting in cerebral infarction	574 (7.50%)	10611 (6.60%)	0.04	574 (7.50%)	527 (6.90%)	0.02
Use of lipid-lower agent						
Statins	2866 (37.34%)	88270 (54.77%)	0.36	2866 (37.34%)	2914 (37.97%)	0.01
Fibrates	223 (2.91%)	5270 (3.27%)	0.02	223 (2.91%)	260 (3.39%)	0.03
Evolocumab	858 (11.18%)	3996 (2.48%)	0.36	858 (11.18%)	845 (11.01%)	<0.01
Alirocumab	358 (4.67%)	1482 (0.92%)	0.26	358 (4.67%)	323 (4.22%)	0.02
Niacin	42 (0.55%)	789 (0.49%)	<0.01	42 (0.55%)	45 (0.59%)	<0.01
Diuretics	1816 (23.67%)	45561 (28.27%)	0.11	1816 (23.67%)	1710 (22.29%)	0.04
SGLT2 inhibitors	425 (5.54%)	10105 (6.27%)	0.03	425 (5.54%)	432 (5.64%)	<0.01
Losartan	998 (13.01%)	23981 (14.88%)	0.05	998 (13.01%)	964 (12.57%)	0.01
**Laboratory**						
Cholesterol (mg/dL)	229.80 +/- 49.90	219.40 +/- 48.80	0.21	229.80 +/- 49.90	228.70 +/- 48.80	0.022
Cholesterol in LDL (mg/dL)	146.70+/- 43.10	137.90 +/- 41.30	0.21	146.70 +/- 43.10	145.50 +/- 42.20	0.029

Cholesterol in HDL (mg/dL)	49.90 +/- 20.20	47.80 +/- 19.50	0.11	49.90 +/- 20.20	49.30 +/- 20.30	0.03
C reactive protein (mg/dL)	10.40 +/- 28.80	16.30 +/- 38.70	0.17	10.40 +/- 28.80	10.50 +/- 28.20	<0.01
Hemoglobin A1C (%)	6.30 +/- 1.30	6.60 +/- 1.60	0.17	6.30 +/- 1.30	6.40 +/- 1.50	0.04
Triglyceride (mg/dL)	161.10 +/- 96.80	161.80 +/- 106.80	<0.01	161.10 +/- 96.80	160.80 +/- 93.30	<0.01
Uric acid (mg/dL)	5.40 +/- 1.20	5.70 +/- 2.00	0.19	5.40 +/- 1.20	5.30 +/- 1.50	0.07

Note: BA, bempedoic acid, EZ, Ezetimibe, and SMD, standardized mean difference.

Supplementary Table 4. Longitudinal changes in laboratory biomarkers at baseline, 3, 6, and 12 months.

Laboratory Parameter	Drug	Baseline (Mean ± SD)	3 months numbers	3 Months (Mean ± SD)	SMD	6 months numbers	6 Months (Mean ± SD)	SMD	12 months numbers	12 Months (Mean ± SD)	SMD
Uric Acid (mg/dL)	BA	5.13 ± 1.05	171	6.22 ± 1.78	0.13	115	6.32 ± 1.80	0.30	116	6.24 ± 1.78	0.16
	EZ	5.09 ± 1.10	66	5.96 ± 2.32		54	5.74 ± 1.98		60	5.97 ± 1.75	
C-Reactive Protein (mg/dL)	BA	10.90 ± 30.60	127	19.29 ± 54.53	0.03	113	16.49 ± 47.02	-0.02	114	10.10 ± 22.34	-0.40
	EZ	10.30 ± 25.30	126	17.58 ± 45.57		133	17.33 ± 48.03		105	29.99 ± 66.52	
LDL-C (mg/dL)	BA	147 ± 43.10	1,823	115.37 ± 47.21	0.15	1,680	119.21 ± 47.89	0.15	1,412	117.70 ± 48.24	0.12
	EZ	145 ± 42.50	1,590	108.46 ± 44.15		1,454	112.22 ± 46.92		1,513	112.28 ± 45.22	
Triglycerides (mg/dL)	BA	160 ± 95.80	1,818	156.80 ± 105.09	0.12	1,689	154.06 ± 94.15	0.11	1,417	148.91 ± 89.55	0.06
	EZ	160 ± 91.30	1,600	144.94 ± 86.48		1,466	144.22 ± 83.41		1,523	143.44 ± 86.53	

Total Cholesterol (mg/dL)	BA	230 ± 49.80	1,743	198.01 ± 53.50	0.17	1,608	201.00 ± 53.45	0.18	1,376	199.53 ± 53.64	0.12
	EZ	228 ± 48.80	1,537	189.33 ± 51.25		1,413	191.64 ± 52.04		1,466	193.22 ± 52.20	
HDL-C (mg/dL)	BA	50.40 ± 20.20	1,832	49.93 ± 20.75	<0.01	1,695	49.40 ± 20.35	0.05	1,420	49.88 ± 20.47	0.05
	EZ	49.90 ± 20.40	1,599	49.88 ± 20.26		1,466	48.36 ± 20.36		1,527	48.85 ± 20.24	
HbA1c (%)	BA	6.30 ± 1.31	993	6.48 ± 1.16	-0.21	1,057	6.45 ± 1.23	-0.19	919	6.43 ± 1.25	-0.10
	EZ	6.33 ± 1.42	912	6.77 ± 1.54		1,019	6.71 ± 1.54		1,061	6.51 ± 1.32	

Note: Laboratory results are presented as mean ± standard deviation (SD) because TriNetX provides only aggregated summary statistics; n indicates the number of patients with at least one laboratory measurement available within the specified follow-up time window. Analyte-specific sample sizes are not available in the TriNetX platform; the number of patients contributing to each timepoint and distribution-based measures (e.g., median [IQR]) are not available. BA, bempedoic acid; EZ, Ezetimibe; SD, Standard Deviation; LDL-C, Low-Density Lipoprotein Cholesterol; HDL-C, High-Density Lipoprotein Cholesterol; HbA1c, hemoglobin A1C; SMD, standardized mean difference.

Supplementary Table 5. Effect of bempedoic acid, compared to the Ezetimibe group, on the risk of hyperuricemia and the treatment of hyperuricemia within the subgroups of the patients.

**Subgroup**	**Risk of hyperuricemia after Propensity-Score Matching Hazard Ratio (95% CI)**	**Risk of UA Treatment After Propensity-Score Matching Hazard Ratio (95% CI)**
Age(yr)		
18-64 yr	2.165 (1.525 - 3.074)	1.418 (0.951 - 2.114)
≥ 65 yr	1.575 (1.227 - 2.023)	0.883 (0.677 - 1.152)
Sex		
male	1.690 (1.271 - 2.248)	1.006 (0.743 - 1.360)
female	1.676 (1.256 - 2.235)	0.804 (0.587 - 1.101)
Smoke		
Yes	1.499 (0.792 - 2.838)	0.685 (0.321 - 1.464)
No	1.964 (1.407 - 2.741)	1.036 (0.724 - 1.482)
BMI		
<27	1.279 (0.741 - 2.208)	0.576 (0.331 - 1.002)
≥27	1.968 (1.391 - 2.785)	1.016 (0.690 - 1.497)
Diabetes mellitus		
Yes	2.104 (1.270 - 3.487)	0.851 (0.487 - 1.489)
No	1.563 (1.053 - 2.320)	0.989 (0.646 - 1.516)
Hypertension		

Yes	1.535 (1.110 - 2.122)	0.865 (0.594 - 1.259)
No	2.095 (0.896 - 4.897)	1.344 (0.601 - 3.004)

Supplementary Figure 1. Study design diagram

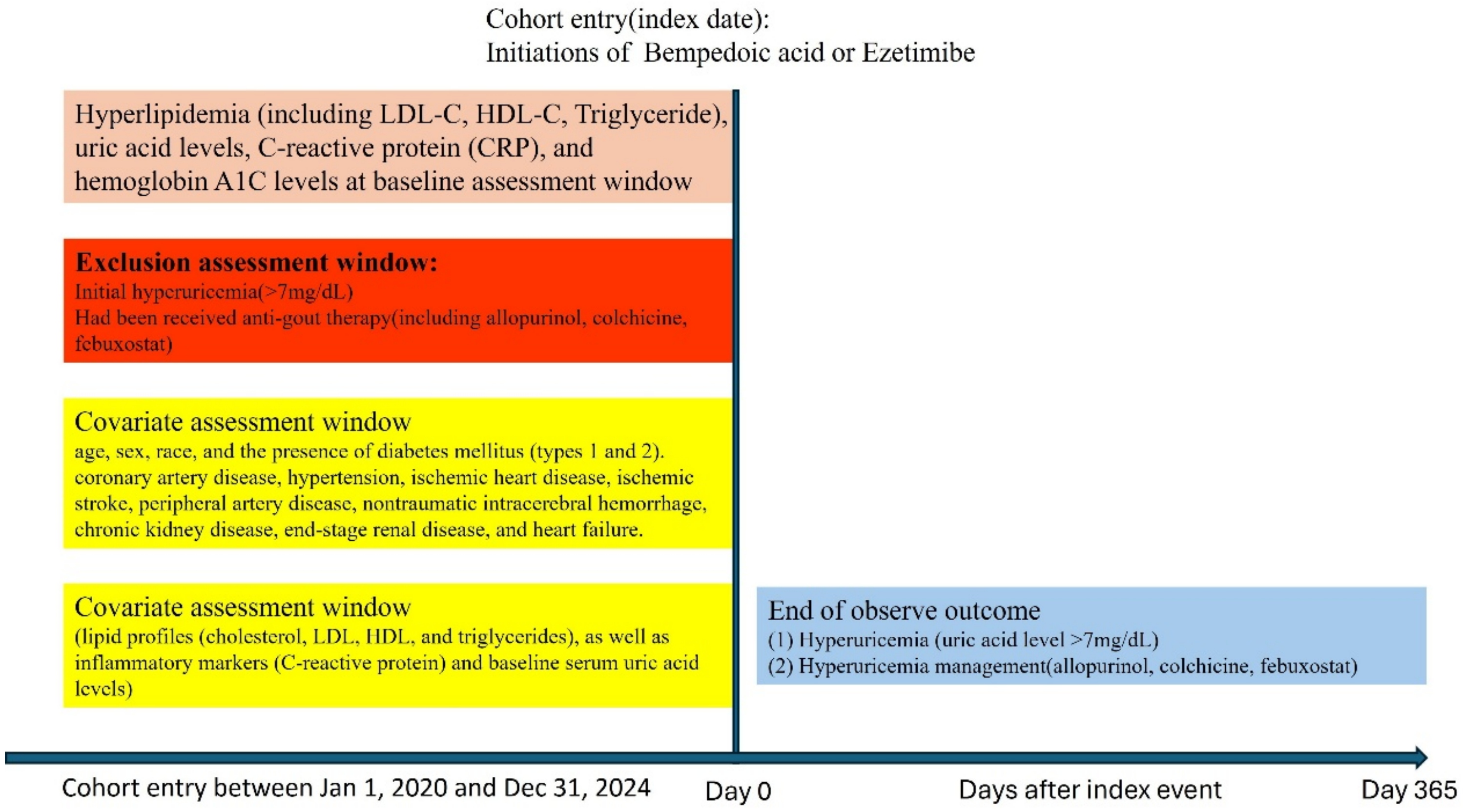

Supplementary Figure 2. Propensity score distributions

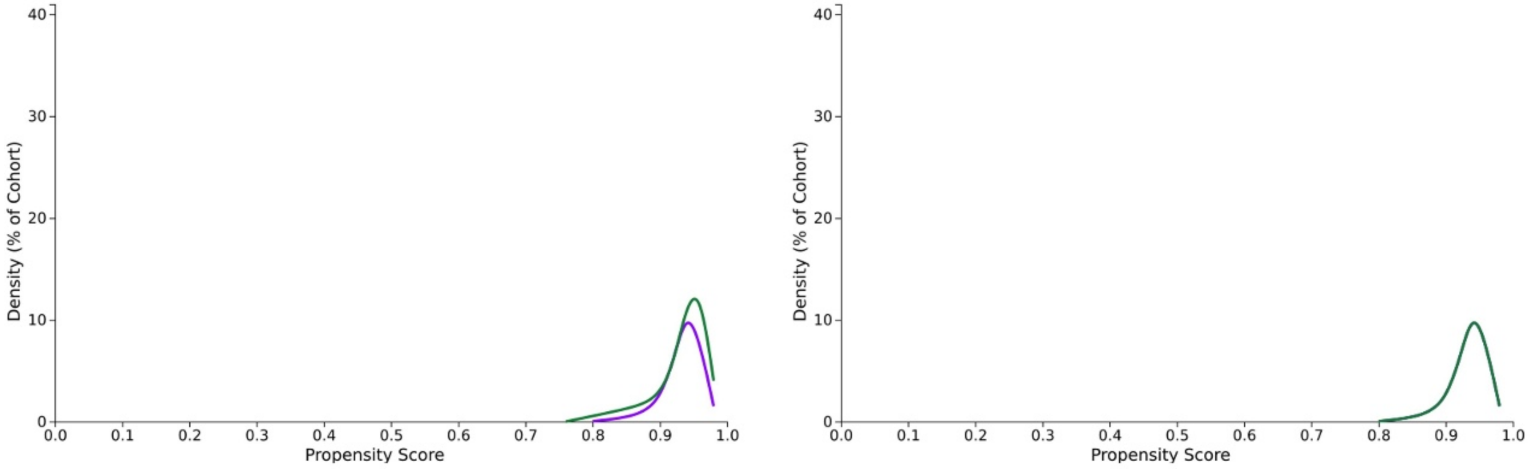

(a) Original cohort

(b)Weighted cohort

(Bempedoic acid - purple, Ezetimibe- green)
