# Supplementary material for: Hyperuricemia risk in bempedoic acid-treated hyperlipidemic patients
Source: Commun Med (Lond). 2026 Apr 1;6:328. doi: 10.1038/s43856-026-01545-2 (PMC13243465; doi:10.1038/s43856-026-01545-2)
Supplement: Supplementary file 2 — Transparent Peer Review file [file 43856_2026_1545_MOESM2_ESM.pdf]

nature portfolio

Peer Review File

Hyperuricemia Risk in Bempedoic Acid-Treated Hyperlipidemic Patients

Corresponding Author: Professor Ping-Yen Liu

This file contains all reviewer reports in order by version, followed by all author rebuttals in order by version.

Version 0:

Reviewer comments:

Reviewer #1

(Remarks to the Author)
This manuscript describes an observational analysis of the risk of developing hyperuricemia with use of bempedoic acid vs. ezetimibe in a large network cohort (7676 patients prescribed bempedoic acid propensity matched to 7676 patients prescribed ezetimibe). The results demonstrate increased serum uric acid, but no increase in the use of anti-gout medications, with use of bempedoic acid compared with use of ezetimibe over 12 months follow-up suggesting that elevated uric acid with bempedoic acid is predominantly mild and asymptomatic. A brief examination of the recent literature suggests that data for this potential adverse effect of bempedoic acid in the "real world" are missing. Therefore, this paper has the potential to add useful information to the available literature. Please see suggestions for modifications below.

1) Suggest changing "An US Collaborative Network Cohort Study" to "A US Collaborative…"
2) Abstract, Page 2, Lines 1-2: part of the 1st sentence appears to be missing; it does not make sense as it is currently written.
3) Abstract, Page 2, Lines 5-7: suggest more clearly stating that propensity matching was used. The phrase "among the matched patients in each treatment group" is not clear, as the matching was not within the groups of patients taking either bempedoic acid or ezetimibe, but rather between them.
4) Abstract, Page 2: suggest including the HRs (95% CIs) for the serum uric acid and anti-gout medication use.
5) Suggest reviewing Wang X, et al. Novel insights into post-marketing AEs associated with bempedoic acid: a comprehensive analysis utilizing the FAERS database. Expert Opin Drug Saf. 2025;Epub ahead of print, for possible citation in the manuscript.
6) Introduction, Page 3, Lines 11-13: suggest using different wording than saying that bempedoic acid is "particularly indicated."
7) Introduction, Page 3, Lines 20-21: the references provided to support the sentence, "…certain lipid-modifying therapies such as niacin, are known to increase serum uric acid levels and precipitate gout in susceptible individuals," are rather outdated and not inclusive. Suggest refining this sentence and including other references, e.g., Leung N, et al. Lowering and raising serum urate levels: Off-label effects of commonly used medications. Mayo Clin Proc. 2022;97(7):1345-1362.
8) Introduction, Page 3, Lines 22-23: suggest adding that ezetimibe does not affect OAT2.
9) Introduction, Page 3, Lines 29-30: suggest including the magnitude of the increase in serum uric acid with bempedoic acid that has been reported in clinical trials, as well as the increase in gout. More detailed reporting of the findings from CLEAR Outcomes, a clinical trial with a similar number of individuals, is needed here and in the Discussion section of the manuscript, which should also include a thorough comparison of those results to the current findings. Notably, the relative increase in the incidence of hyperuricemia is present in the bempedoic acid group for this cohort (94%) compared to CLEAR Outcomes (95%), but that is not accompanied by the 48% relative increase in gout that was reported CLEAR Outcomes.
10) Introduction, Page 4, Lines 11-15: this statement of the results of the current investigation are out of place in the Introduction section.
11) Methods, Page 4, Line 12: suggest changing the subheading title from "Results and follow-up" to "Outcomes of interest."
12) Methods, Page 5, Lines 13-15: as noted, newer guidelines, including those from the American College of Rheumatology in 2020, define hyperuricemia as >6.8 mg/dL. Suggest citing the guidelines where the lower threshold is mentioned. Also, what was the cut point used for defining hyperuricemia in the clinical trials of bempedoic acid?
13) Methods, Page 5, Lines 17-19: it appears that in order to be defined as "a clinically significant episode of hyperuricemia" in this investigation that the presence of elevated uric acid and the initiation of anti-gout therapy were both required. Suggest including information about patients who were discordant, i.e., had elevated uric acid but no use of anti-gout therapy; or had use of anti-gout therapy but uric acid was not elevated. Also, suggest listing the specific anti-gout medications that were identified for this investigation.

14) Methods, Pages 5-6, Lines 28-31: were the covariates described here (e.g., age, sex, race, comorbidities, etc.) further adjusted in the analyses beyond their use in the propensity matching?
15) Methods, Page 6, Lines 4-14: suggest more details are needed regarding the statistical analyses. Primary, subgroup, and sensitivity analyses were done, but there are few details in the Methods about what these were. Also, include a statement of how the hazard ratios were calculated, i.e., Cox proportional hazards. For the secondary outcomes, was it a requirement for inclusion that the patients had values for these at each of the measurement timepoints (3, 6, and 12 months)? Table 3 does not include the number of patients included in those analyses, so it is not clear. Also, there were apparently no statistical comparisons of the secondary outcomes. Please provide rationale for this.
16) Results, Page 6, Line 17: Figure 1 does not really illustrate the propensity score matching process, rather it simply shows the number of subjects excluded to create the propensity score matched groups of patients.
17) Results, Page 7, Section 3.2 and Table 1: use of other lipid-lowering agents by the patients, as well as other agents with potential to affect serum uric acid and gout, should be reported.
18) Results, Page 8, Section 3.3.2: the second paragraph in this section that provides an interpretation of the figures for the changes in secondary outcomes over time is unnecessary. The generalizations of comparable, minimal, similar, etc., do not always align with the appearance of the figures, in part because of the scales of the graphs to include the large error bars. Suggest instead that the text simply refer to Figure 2 which shows the results for these measurements over time, and then the actual values that are reported in Table 3 can be discussed in more detail (as is done in the next paragraph).
19) Results, Page 8, Lines 23-24: the sentence, "During follow-up, both bempedoic acid and ezetimibe exhibited comparable effects on lipid and metabolic parameters, although they exhibited distinct variations in specific biomarkers." appears to make 2 difference conclusions. Suggest revising for clarity.
20) Results, Page 8, Lines 28-29: the statement that for total and LDL-C, "the degree of reduction was slightly more consistent in the bempedoic acid cohort" is too general and does not reflect the fact that both total and LDL-C were lower after 12 months with ezetimibe than with bempedoic acid. Also, suggest revising the similar statement in the Discussion (Page 10, Lines 14-17).
21) Results, Page 8, Lines 29-30: suggest refining the description of the CRP results. Currently it says that CRP "decreased more markedly in patients who received bempedoic acid." According to Table 3, the baseline levels were 10.90 and 10.30 for bempedoic acid and ezetimibe, respectively, and at 12 months were 10.10 and 29.99, respectively. However, at 3 months, values with bempedoic acid were higher than with ezetimibe, and at 6 months they were relatively similar. Also, suggest revising the similar statement in the Discussion (Page 10, Lines 17-18). Please check the units reported for CRP; they are listed in Table 3 as mg/L but based on the values are likely mg/dL. Also, CRP is typically not normally distributed and should be reported as median and IQR limits instead of means and SD.
22) Results, Page 9, Line 5-13: as noted earlier, a description of the subgroup and sensitivity analyses is needed in the Methods section so that the reader can understand statements in the Results that refer generally to those subgroups, e.g., "…were largely consistent with the main findings…"
23) Discussion, Page 11, Lines 17-19: the point is made here that there was substantial variability in the CRP levels, which should also be recognized in the description of the Results.
24) Discussion: While the authors have briefly mentioned some limitations of their analyses, a stronger acknowledgement of these limitations is needed. Suggest recognizing the possibility for a type I error (longer follow-up may have demonstrated a significant effect). Also, as mentioned earlier in the comments, suggest carefully comparing these results to those from CLEAR Outcomes, including a direct comparison of the magnitude of the incidence for hyperuricemia and gout, and considering differences in the way these were defined. Furthermore, the likelihood that some patients with mild gout symptoms may have received anti-inflammatory and/or analgesic drugs for their symptoms does not appear to be captured by this analysis that examined use of "anti-gout medications." Thus, the true incidence of symptomatic gout has likely been underestimated. Given the shorter period of follow-up compared to CLEAR Outcomes, could the lack of association between bempedoic acid use and incident gout, despite greater incidence of hyperuricemia, be simply due to a type II statistical error?
25) Conclusions, Page 12, Line 1: instead of indicating that routine UA surveillance or intervention "is not acceptable" suggest writing it as "may not be necessary."
26) Figure 2: units for the laboratory variables are missing from the figure; also, include the number of patients.
27) Table 3: the number of patients in each analysis are missing; also, triglycerides and CRP are not usually normally distributed and should be reported as medians and IQR limits instead of means and SDs.

Minor comments:
1) Abstract: suggest defining abbreviations at their first use (unless the journal allows use of certain abbreviations without definitions).
2) Throughout the Methods, analyzes should be spelled analyses.
3) Methods, Page 5, Line 27 and Discussion, Page 11, Line 8: suggest replacing "confusion" with "confounding."
4) Discussion, Page 10, Line 25: suggest revising to "Our real-world data support the findings of the clinical trials…"
5) Table 1: please include the units for the laboratory values; please use Arabic numerals for diabetes, ie, Type 1 and Type 2 diabetes mellitus.

Reviewer #2

(Remarks to the Author)
The manuscript is novel and useful in that it provides data around whether the known risks of increased serum uric acid levels with bempedoic acid results in increased use of uric acid lowering medications within the real-world setting based on a large claims dataset. The information is helpful by providing practical use information on bempedoic acid for the prescriber.

Major comments:

1) General:
a) Bempedoic acid is also available as a combination tablet with ezetimibe. Did you exclude patients on combination use?
b) Include the manuscript Ray et al, within the Discussion section that was recently published in JACC: advances which focuses on use of uric acid lowering medications in CLEAR Outcomes trial.
c) Did you look at gout in over the index period? If not, why not since the risk of elevated serum uric acid is gout.
2) Page 4, lines 18-20: Please confirm that this study was conducted using the global TriNetX network (the true "federated global network") or the more commonly used US TriNetx dataset. The n's and demographics are more representative of the US dataset.
3) Page 4, lines 29-30:
Conceptually, not sure this makes sense. You can't mimic a RCT with a retrospective observational database study, as there is no intervention. I think what they mean is that they had pre- and post-index observation periods. It is misleading to say that they mimicked an RCT. Perhaps they are referring to the propensity score matching process? but that only is trying to mimic the randomization aspect of an RCT.
4) Page 5, lines 9-11: What were the years of index rx identification / data collection?
- What was the pre-index observation period? 12 mos pre? I believe it said 12 mos post
- How were patients determined to be "New users" of bempedoic acid or ezetimibe (related to duration of the pre-index observation period)
- What about baseline and pre-index rx utilization?
5) Page 6, lines 8-10: What was the match? 1:1, 1:2? how were patients matched?
What were the 2 groups matched on? all of the baseline demographic and clinical characteristics? only select ones?
6) Page 5, lines 2-5: was past medical history of gout excluded?

Minor comments:
7) The manuscript needs to be reviewed carefully for grammatical edits. Examples just in the abstract include page 2, lines 2-3; line 7.
8) Page 3, Lines 10-13: bempedoic acid is approved and the indication referenced should be revised to be current (it is indicated beyond ASCVD or HeFH now).
9) Page 3, lines 23-24: clarify that what is not understood is any implications to increased serum uric acid in the real-world setting. Check for this clarification throughout manuscript.
10) Page 4, lines 11-15: Remove results from the introduction section.
11) Page 6, line 2: uric acid is not an inflammatory marker and should be updated.
12) Page 6, lines 12-14: exploratory analyses referenced but not included- please address.
13) Page 8, line 5: describe how anti-gout medications were defined.
14) Page 9, lines 27-28: incidence of gout numbers from CLEAR Outcomes is incorrect. Data is 3.1% vs 2.1%
15) Figure 2 for CRP. ZE should be EZ. Also, need to add n's at the time points so you know how many patients data is available.
17) Table 3: add n's,
18) Page 18, for the laboratory values, need to add units. Use LDL-C vs "Cholesterol in LDL"- same for HDL.

Version 1:

Reviewer comments:

Reviewer #1

(Remarks to the Author)
Thank you for addressing the previous comments. The manuscript is much improved, but there are a few responses that are somewhat unclear and/or incompletely address the comment. Please see below.
1. To address the previous request to include information about patients who were discordant (i.e., had elevated uric acid but no use of anti-gout therapy OR had use of anti-gout therapy but uric acid was not elevated), the authors added to the Methods section that discordant cases were analyzed separately. However the results of this discordant analysis do not appear to have been incorporated into the Results section of the manuscript.
2. Previously, it was suggested that the authors clarify the following statement about total and LDL-C responses: “the degree of reduction was slightly more consistent in the bempedoic acid cohort.” While the authors indicate in their rebuttal that they “corrected these statements in the Results and Discussion sections to accurately represent the numerical data, indicating that total cholesterol and LDL-C levels were slightly lower after 12 months in the ezetimibe cohort compared with the bempedoic acid cohort” this does not seem to be the case. On Page 11, Lines 304-305 of the Results section still state, “Although both therapies led to reductions in LDL-C and total cholesterol, the degree of reduction was slightly more consistent in the bempedoic acid cohort.” Similarly, Page 13, Lines 371-372 state, “both bempedoic acid and ezetimibe were similarly effective in lowering LDL-C and total cholesterol, and the bempedoic acid cohort showed slightly more consistent reductions.”

3. Another reviewer had commented that bempedoic acid is also available as a combination tablet with ezetimibe and asked if subjects using the combination were excluded. In their response to this comment the authors provide hazard ratios for developing hyperuricemia and for use of antigout therapy “in the bempedoic acid group” but it isn’t clear how this response addresses the comment regarding a combination bempedoic acid/ezetimibe tablet.
4. The authors have explained in their responses and in the manuscript that they could not provide the numbers of subjects included for the secondary outcomes because the TriNetX analytics platform provides aggregated summary statistics for each time point but does not disclose the number of patients who contributed. However, in some of their responses, the authors indicate that they have updated the tables and figures to include “the number of patients (n) at each time point for all laboratory variables, providing transparency regarding the availability of data.” Please check throughout the manuscript and clarify.

Reviewer #2

(Remarks to the Author)
The authors have addressed a number of questions but a few questions/changes need to be addressed based on the updated text.
This study as described in the current publication does provide new and useful information for the prescriber using bempedoic acid.

1. Abstract, Lines 4-5: “However, its effects on serum uric acid (UA) are not yet fully defined, with potential implications for the risk of gout in clinical practice.”
The effects of bempedoic acid on serum uric acid have been studied in 4 phase 3 randomized controlled trials, and one phase 3 cardiovascular outcomes trial which has consistently demonstrated BA increases serum uric acid by 0.8 mg/dL compared to placebo. What you are assessing are the effects of bempedoic acid on serum acid with real world use. Thus, you should appropriately reword this sentence to something like “However data regarding the effects of bempedoic acid on serum uric acid levels and the use of anti-gout therapy with real world use is lacking.”

2. Abstract lines 14-17 states “These findings suggest that while bempedoic acid may elevate serum UA, its clinical impact on gout treatment needs appears limited, supporting its continued use in patients requiring further LDL-C lowering while emphasizing the need for monitoring in individuals at risk of hyperuricemia.’
Your data doesn’t support an emphasis on monitoring uric acid levels in individuals taking bempedoic acid. Further, the guidance in the label for bempedoic acid (Nexletol or Nexlizet) suggests to assess serum acid when clinically indicated. The data supporting anti-gout medication use not being different than the ezetimibe cohort suggests that the serum acid increases weren’t leading to differences in treatment between the two groups. The text should be updated to reflect the data.

3. Introduction, page 4, lines 10-16: bempedoic acid is also approved in the US, EU and other countries to lower cardiovascular risk. This should be incorporated into the text.

4. Plain Language Summary, Lines 8-9 which state “These findings may help doctors choose cholesterol treatments for patients who are at risk of gout” should be updated to reflect the data. The data doesn’t help doctors choose when treatment for patients at risk of gout. Please see other comments and update to reflect the data from this analysis.

5. Introduction, page 4, line 27: “However, the clinical relevance of this effect is not yet understood.” The clinical relevance of the increase in uric acid is known from clinical trials- it is associated with ~1% increased risk of gout compared to placebo. What is not known is the increase in serum uric acid in clinical practice and the use of anti-gout medications. This sentence needs to be adjusted according. Page 5, Lines 1-9 which is new text is better written and repetitive of the text on page 4. This should be adjusted accordingly.

6. Study Design, page 6: repetition in lines 2-4 with 11-13. This needs to be addressed.

7. Discussion, page 12, line 24: I have not seen anything to suggest the hyperuricemia events in the clinical program were adjudicated. They were investigator-reported adverse events based on the publications. Please adjust wording accordingly.

8. Discussion page 13, lines 28-30: bempedoic acid has been reported to reduce median levels of hsCRP between 20-30%. The data should be stated with supporting publications.

9. Strengths and Limitations: I think you need to clearly indicate as an limitation that the incidence of the anti-gout medications cannot be construed as gout per se as patients may not have treated gout, may receive treatment outside of this claims database, etc. Did you look at events of gout per se?

10. Table 3: CRP is not normally distributed and should be reported in median with IQR.

Version 2:

Reviewer comments:

Reviewer #1

(Remarks to the Author)
Thank you for addressing all of the previous comments.

Reviewer #2

(Remarks to the Author)
In my opinion, the authors have appropriately addressed all comments and the manuscript is improved. I recommend approving the manuscript for the next steps towards publication.

Open Access This Peer Review File is licensed under a Creative Commons Attribution 4.0 International License, which permits use, sharing, adaptation, distribution and reproduction in any medium or format, as long as you give appropriate credit to the original author(s) and the source, provide a link to the Creative Commons license, and indicate if changes were made.
In cases where reviewers are anonymous, credit should be given to 'Anonymous Referee' and the source.
The images or other third party material in this Peer Review File are included in the article's Creative Commons license, unless indicated otherwise in a credit line to the material. If material is not included in the article's Creative Commons license and your intended use is not permitted by statutory regulation or exceeds the permitted use, you will need to obtain permission directly from the copyright holder.
To view a copy of this license, visit https://creativecommons.org/licenses/by/4.0/

Response to Reviewers Comments

Reviewer1:

The authors sincerely thank the reviewer for the thoughtful and constructive comments. Each point has been carefully addressed below, and the manuscript has been revised accordingly to improve clarity, accuracy, and transparency.

Major Comments:

Comment 1:

Suggest changing "An US Collaborative Network Cohort Study" to "A US Collaborative…"

Response:

The authors thank the reviewer for this valuable suggestion. In the revised manuscript, we have corrected the title to "Hyperuricemia Risk in Bempedoic Acid-Treated Hyperlipidemic Patients: A US Collaborative Network Cohort Study."

Comment 2:

Abstract, Page 2, Lines 1-2: part of the 1st sentence appears to be missing; it does not make sense as it is currently written.

Response:

We thank the reviewer for pointing out this missing part. The first sentence of the Abstract has been revised to clearly introduce the study background and objective.

In the revised manuscript (Page 2, Lines 2-5), the corrected text reads as follows: "Bempedoic acid, an adenosine triphosphate citrate lyase inhibitor, is approved to lower low-density lipoprotein cholesterol (LDL-C) cholesterol in patients with hyperlipidemia requiring additional reduction in lipids. However, its effects on serum uric acid (UA) are not yet fully defined, with potential implications for the risk of gout in clinical practice."

Comment 3:

Abstract, Page 2, Lines 5-7: suggest more clearly stating that propensity matching was used. The phrase "among the matched patients in each treatment group" is not clear, as the matching was not within the groups of patients taking either bempedoic acid or ezetimibe, but rather between them.

Response:

We appreciate the reviewer's valuable clarification and have revised the Abstract to more accurately describe the propensity score matching process.

In the revised manuscript (Page 2, Lines 7-9), the sentence now reads as follows: "Using propensity score matching between treatment groups, 7,676 patients receiving bempedoic acid were matched to 7,676 patients receiving ezetimibe."

Comment 4:

Abstract, Page 2: suggest including the HRs (95% CIs) for the serum uric acid and anti-gout medication use.

Response:

We appreciate the reviewer's valuable suggestion and have revised the Abstract to include the hazard ratios (HRs) with 95% confidence intervals (CIs) for serum uric acid and anti-gout medication use.

In the revised manuscript (Page 2, Lines 9-12), the sentence now reads as follows: "The incidence of hyperuricemia was significantly higher in the bempedoic acid group (Hazard ratio (HR): 1.94; 95% confidence interval (CI): 1.58 - 2.37; $p < 0.01$) compared to the ezetimibe group. However, no significant differences were observed in the initiation of anti-gout therapy (HR: 1.06; 95% CI: 0.86 - 1.29; $p = 0.59$).

Comment 5:

Suggest reviewing Wang X, et al. Novel insights into post-marketing AEs associated with bempedoic acid: a comprehensive analysis utilizing the FAERS database. Expert Opin Drug Saf. 2025;Epub ahead of print, for possible citation in the manuscript.

Response:

The authors thank the reviewer for this valuable suggestion. We have reviewed the recommended article by Wang et al. (2025) and incorporated it into the Discussion to better contextualize our findings with post-marketing pharmacovigilance data.

In the revised manuscript (Page 13, Lines 16-19), the sentence now reads as follows: "Our findings are consistent with recent pharmacovigilance data from the FDA Adverse Event Reporting System (FAERS), which also identified hyperuricemia and gout among the most frequently reported metabolic adverse events associated with bempedoic acid use, though the overall incidence remained low.[23]"

Reference:

23. Wang X, Liu Y, Han H, Ma J, Tian N, Zhu R, Shi X, Jin J, Zhou H. Novel insights into post-marketing AEs associated with bempedoic acid: a comprehensive analysis utilizing the FAERS database. Expert Opin Drug Saf. 2025 Feb 19:1-8.

Comment 6:

Introduction, Page 3, Lines 11-13: suggest using different wording than saying that bempedoic acid is "particularly indicated."

Response:

We understand the reviewer's thoughtful suggestion. To ensure the wording aligns with current prescribing indications and maintains a neutral scientific tone, we have revised the sentence accordingly.

In the revised manuscript (Page 4, Lines 13-16), the sentence now reads as follows: "It is used primarily in patients with atherosclerotic cardiovascular disease (ASCVD) or heterozygous familial hypercholesterolemia who are unable to tolerate statins or achieve adequate LDL-C reduction with statin therapy alone [8–10]."

Comment 7:

Introduction, Page 3, Lines 20-21: the references provided to support the sentence, "…certain lipid-modifying therapies such as niacin, are known to increase serum uric acid levels and precipitate gout in susceptible individuals," are rather outdated and not inclusive. Suggest refining this sentence and including other references, e.g., Leung N, et al. Lowering and raising serum urate levels: Off-label effects of commonly used medications. Mayo Clin Proc. 2022;97(7):1345-1362.

Response:

The authors thank the reviewer for this valuable suggestion. We have revised the sentence to improve clarity and updated the references to include the more recent and comprehensive review by Leung et al. (2022), which summarizes the effects of various medications which including lipid-lowering agents on serum urate levels.

In the revised manuscript (Page 4, Lines 22-24), the sentence now reads as follows: "Importantly, some lipid-modifying therapies, particularly niacin, have been shown to elevate serum uric acid levels and may precipitate gout in predisposed individuals [15,16,17]."

Reference:

17. *Leung N, Yip K, Pillinger MH, Toprover M. Lowering and Raising Serum Urate Levels: Off-Label Effects of Commonly Used Medications. Mayo Clin Proc. 2022 Jul;97(7):1345-1362.*

Comment 8:

Introduction, Page 3, Lines 22-23: suggest adding that ezetimibe does not affect OAT2.

Response:

The authors thank the reviewer for this valuable suggestion. To provide a mechanistic contrast between the study drugs, we have clarified that ezetimibe does not influence OAT2 or urate metabolism.

In the revised manuscript (Page 4, Lines 25-27), the sentence now reads as follows: "Bempedoic acid can increase serum UA by inhibiting organic anion transporter 2 (OAT2) in proximal tubules, thereby reducing urate clearance, whereas ezetimibe does not affect OAT2 activity or urate metabolism[18]."

Comment 9:

Introduction, Page 3, Lines 29-30: suggest including the magnitude of the increase in serum uric acid with bempedoic acid that has been reported in clinical trials, as well as the increase in gout. More detailed reporting of the findings from CLEAR Outcomes, a clinical trial with a similar number of individuals, is needed here and in the Discussion section of the manuscript, which should also include a thorough comparison of those results to the current findings. Notably, the relative increase in the incidence of hyperuricemia is present in the bempedoic acid group for this cohort (94%) compared to CLEAR Outcomes (95%), but that is not accompanied by the 48% relative increase in gout that was reported CLEAR Outcomes.

Response:

The authors thank the reviewer for this thoughtful suggestion. We have revised the introduction and discussion to include the magnitude of serum uric acid and gout increases observed in major randomized controlled trials, particularly the CLEAR Outcomes trial, and to contrast those findings with our real-world results.

In the revised manuscript (Page 5, Lines 1-9), the sentence now reads as follows: "Although randomized trials have quantified the biochemical impact of bempedoic acid on serum UA, including CLEAR Harmony and CLEAR Outcomes, bempedoic acid was

associated with a mean serum UA increase of approximately 0.76 mg/dL and a relatively increased risk of gout compared with placebo (3.1% vs. 2.1%). These findings highlight the potential for urate-related adverse events; however, trial participants were highly selected and closely monitored, limiting the applicability of these results to routine clinical practice. Although bempedoic acid is known to increase serum UA levels, the clinical implications of this effect in real-world settings remain uncertain."

As reported in Nissen et al., 2023, CLEAR Outcomes trial:

Nissen SE, Lincoff AM, Brennan D, Ray KK, Mason D, Kastelein JJP, Thompson PD, Libby P, Cho L, Plutzky J, Bays HE, Moriarty PM, Menon V, Grobbee DE, Louie MJ, Chen CF, Li N, Bloedon L, Robinson P, Horner M, Sasiela WJ, McCluskey J, Davey D, Fajardo-Campos P, Petrovic P, Fedacko J, Zmuda W, Lukyanov Y, Nicholls SJ; CLEAR Outcomes Investigators. Bempedoic Acid and Cardiovascular Outcomes in Statin-Intolerant Patients. N Engl J Med. 2023 Apr 13;388(15):1353-1364.

Comment 10:

Introduction, Page 4, Lines 11-15: this statement of the results of the current investigation are out of place in the Introduction section.

Response:

The authors thank the reviewer for this important observation. In the revised manuscript, we have revised the introduction section to focus completely on the study rationale and objectives, removing content related to study results.

Comment 11:

Methods, Page 4, Line 12: suggest changing the subheading title from "Results and follow-up" to "Outcomes of interest."

Response:

The authors thank the reviewer for this helpful suggestion. In the revised manuscript, the Methods section subheading has been updated from "Results and follow-up" to "2.3 Outcomes of interest" to more accurately describe the primary and secondary study endpoints.

Comment 12:

Methods, Page 5, Lines 13-15: as noted, newer guidelines, including those from the American College of Rheumatology in 2020, define hyperuricemia as >6.8 mg/dL. Suggest

citing the guidelines where the lower threshold is mentioned. Also, what was the cut point used for defining hyperuricemia in the clinical trials of bempedoic acid?

Response:

The authors thank the reviewer for this important comment. In the revised manuscript, the Methods section has been updated to cite the 2020 American College of Rheumatology (ACR) guidelines, which define hyperuricemia as a serum urate level >6.8 mg/dL. We also clarified that our chosen cutoff (>7.0 mg/dL) aligns with the definition used in major bempedoic acid clinical trials (CLEAR Harmony, CLEAR Wisdom, and CLEAR Outcomes).

In the revised manuscript (Page 6, Lines 21-27), the sentence now reads as follows: "The primary outcome of interest was the incidence of hyperuricemia, defined as a UA level >7.0 mg/dL, consistent with the cutoff used in pivotal bempedoic acid trials (CLEAR Harmony, CLEAR Wisdom, and CLEAR outcomes). This threshold corresponds closely to the point of urate supersaturation in plasma. Although the 2020 American College of Rheumatology (ACR) guidelines[21] define hyperuricemia as >6.8 mg/dL, the theoretical limit of solubility of urate under physiological conditions, most large-scale epidemiological and interventional studies have continued to adopt 7.0 mg/dL for clinical relevance and comparability across datasets."

Reference:

21. FitzGerald JD, Dalbeth N, Mikuls T, Brignardello-Petersen R, Guyatt G, Abeles AM, Gelber AC, Harrold LR, Khanna D, King C, Levy G, Libbey C, Mount D, Pillinger MH, Rosenthal A, Singh JA, Sims JE, Smith BJ, Wenger NS, Bae SS, Danve A, Khanna PP, Kim SC, Lenert A, Poon S, Qasim A, Sehra ST, Sharma TSK, Toprover M, Turgunbaev M, Zeng L, Zhang MA, Turner AS, Neogi T. 2020 American College of Rheumatology Guideline for the Management of Gout. Arthritis Care Res (Hoboken). 2020 Jun;72(6):744-760.

Comment 13:

Methods, Page 5, Lines 17-19: it appears that in order to be defined as "a clinically significant episode of hyperuricemia" in this investigation that the presence of elevated uric acid and the initiation of anti-gout therapy were both required. Suggest including information about patients who were discordant, i.e., had elevated uric acid but no use of

anti-gout therapy; or had use of anti-gout therapy but uric acid was not elevated. Also, suggest listing the specific anti-gout medications that were identified for this investigation.

Response:

The authors thank the reviewer for this thoughtful suggestion. In the revised manuscript, we have clarified in the Methods section that a "clinically significant episode of hyperuricemia" was defined as the coexistence of an elevated serum uric acid level (>7.0 mg/dL) and the initiation of anti-gout therapy. To address the reviewer's comment, we have also included information on discordant cases, patients who exhibited elevated uric acid without anti-gout therapy, and those who initiated anti-gout treatment without documented hyperuricemia. Furthermore, we have specified all anti-gout medications included in the analysis.

Also, in the revised manuscript, the Methods and Results sections now state that discordant cases were analyzed separately. Specifically, among patients who developed hyperuricemia without subsequent anti-gout therapy during follow-up, 185 were in the bempedoic acid group and 86 were in the ezetimibe group. Conversely, among patients who initiated anti-gout therapy without recorded hyperuricemia, 134 were in the bempedoic acid group and 121 were in the ezetimibe group.

In the revised manuscript (Page 7, Lines 2-8), the sentence now reads as follows: "Two complementary definitions were applied. Biochemical hyperuricemia was defined as serum UA >7.0 mg/dL, while clinically significant hyperuricemia required both elevated UA and the initiation of anti-gout therapy during follow-up. Patients with elevated UA but no anti-gout therapy were categorized as having asymptomatic hyperuricemia, whereas those who initiated anti-gout therapy without an elevated UA were considered discordant and analyzed separately. Anti-gout medications included xanthine oxidase inhibitors (allopurinol, febuxostat), and colchicine."

Comment 14:

Methods, Pages 5-6, Lines 28-31: were the covariates described here (e.g., age, sex, race, comorbidities, etc.) further adjusted in the analyses beyond their use in the propensity matching?

Response:

The authors thank the reviewer for this important clarification. In our primary analysis, the covariates including age, sex, race, comorbidities, and concurrent medications are listed in Table 1 were incorporated into the propensity score model for 1:1 matching between treatment groups. Following matching, the baseline characteristics were well balanced across cohorts; therefore, no additional covariate adjustment was applied in the outcome models. This approach is consistent with standard analytic practices for propensity score-matched cohort studies.

In the revised manuscript (Page 7, Lines 24-31 and page 8, line 1-13), the sentence now reads as follows: "Continuous variables were summarized as means with standard deviations (mean ± SD), and categorical variables were expressed as absolute counts and percentages. To ensure baseline comparability between treatment groups, a propensity score matching was performed prior to the primary, subgroup, and sensitivity analyses. Propensity scores were estimated using multivariable logistic regression that included age, sex, race, baseline comorbidities (e.g., hypertension, diabetes mellitus, chronic kidney disease, coronary artery disease, heart failure), and concurrent cardiovascular medications. Balance between matched cohorts was assessed using the absolute standardized mean difference (SMD), with an SMD <0.1 indicating adequate balance. After matching, no further covariate adjustment was applied in the primary analysis, as covariate balance had been achieved.

The primary analysis estimated the risk of hyperuricemia incident among patients receiving bempedoic acid versus ezetimibe using Cox proportional hazards regression, with results expressed as Hazard ratios (HRs) and 95% CIs. To evaluate the consistency of our findings, we conducted prespecified subgroup analyses across clinically relevant categories that could potentially modify the relationship between bempedoic acid use and hyperuricemia. Subgroups included age (<65 vs. ⩾65 years), sex, baseline UA status (normal vs. elevated), presence of diabetes mellitus, hypertension, chronic kidney disease, and baseline use of diuretics. These factors were selected based on their known associations with UA metabolism and gout risk. For each subgroup, HRs and 95% CIs for the outcomes of interest, incident hyperuricemia and initiation of anti-gout therapy, were re-estimated using Cox proportional hazards models within the propensity score-matched cohort."

Comment 15:

Methods, Page 6, Lines 4-14: suggest more details are needed regarding the statistical analyses. Primary, subgroup, and sensitivity analyses were done, but there are few details in the Methods about what these were. Also, include a statement of how the hazard ratios were calculated, i.e., Cox proportional hazards. For the secondary outcomes, was it a requirement for inclusion that the patients had values for these at each of the measurement timepoints (3, 6, and 12 months)? Table 3 does not include the number of patients included in those analyses, so it is not clear. Also, there were apparently no statistical comparisons of the secondary outcomes. Please provide rationale for this.

Response:

The authors thank the reviewer for this valuable feedback. In the revised manuscript, we have expanded the Methods section to provide a clearer description of the statistical analyses conducted. We now specify that hazard ratios (HRs) and 95% confidence intervals (CIs) were estimated using Cox proportional hazards models for time-to-event outcomes. Additionally, we have clarified that the secondary biochemical outcomes including LDL-C, HDL-C, triglycerides, total cholesterol, CRP, HbA1c, and serum uric acid, only patients with available laboratory data at each specific follow-up timepoint (3, 6, or 12 months) were included in the corresponding analysis. These analyses were descriptive and exploratory; thus, formal statistical comparisons were not conducted due to variable data completeness and the potential for selection bias resulting from non-random laboratory testing patterns. We have also included the number of patients analyzed for each secondary outcome in Table 3 to enhance transparency.

In the revised manuscript (Page 8, Lines 3-19 and Table-3), the sentence now reads as follows: "The primary analysis estimated the risk of hyperuricemia incident among patients receiving bempedoic acid versus ezetimibe using Cox proportional hazards regression, with results expressed as Hazard ratios (HRs) and 95% CIs. To evaluate the consistency of our findings, we conducted prespecified subgroup analyses across clinically relevant categories that could potentially modify the relationship between bempedoic acid use and hyperuricemia. Subgroups included age (<65 vs. ≥65 years), sex, baseline UA status (normal vs. elevated), presence of diabetes mellitus, hypertension, chronic kidney disease, and baseline use of diuretics. These factors were selected based on their known associations with UA metabolism and gout risk. For each subgroup, HRs and 95% CIs for the outcomes

of interest, incident hyperuricemia and initiation of anti-gout therapy, were re-estimated using Cox proportional hazards models within the propensity score-matched cohort.

Secondary biochemical outcomes, including changes in LDL-C, HDL-C, triglycerides, total cholesterol, CRP, HbA1c, and UA were evaluated at 3, 6, and 12 months. Patients were included in each time point-specific analysis only if the corresponding laboratory values were available. Given the variability in the frequency of tests and the missing data between sites, these results were summarized descriptively rather than compared statistically between treatment groups. This approach minimized potential bias related to non-random laboratory measurement."

Table 3. Longitudinal changes in laboratory biomarkers at baseline, 3, 6, and 12 months.

**Laboratory Parameter**	**Drug**	**Baseline (Mean ± SD)**	**3 months numbers**	**3 Months (Mean ± SD)**	**SMD**	**6 months numbers**	**6 Months (Mean ± SD)**	**SMD**	**12 months numbers**	**12 Months (Mean ± SD)**	**SMD**
Uric Acid (mg/dL)	BA	5.13 ± 1.05	171	6.22 ± 1.78	0.13	115	6.32 ± 1.80	0.30	116	6.24 ± 1.78	0.16
	EZ	5.09 ± 1.10	66	5.96 ± 2.32		54	5.74 ± 1.98		60	5.97 ± 1.75	
C-Reactive Protein (mg/dL)	BA	10.90 ± 30.60	127	19.29 ± 54.53	0.03	113	16.49 ± 47.02	-0.02	114	10.10 ± 22.34	-0.40
	EZ	10.30 ± 25.30	126	17.58 ± 45.57		133	17.33 ± 48.03		105	29.99 ± 66.52	
LDL-C (mg/dL)	BA	147 ± 43.10	1,823	115.37 ± 47.21	0.15	1,680	119.21 ± 47.89	0.15	1,412	117.70 ± 48.24	0.12
	EZ	145 ± 42.50	1,590	108.46 ± 44.15		1,454	112.22 ± 46.92		1,513	112.28 ± 45.22	
Triglycerides (mg/dL)	BA	160 ± 95.80	1,818	156.80 ± 105.09	0.12	1,689	154.06 ± 94.15	0.11	1,417	148.91 ± 89.55	0.06
	EZ	160 ± 91.30	1,600	144.94 ± 86.48		1,466	144.22 ± 83.41		1,523	143.44 ± 86.53	

Total Cholesterol (mg/dL)	BA	230 ± 49.80	1,743	198.01 ± 53.50	0.17	1,608	201.00 ± 53.45	0.18	1,376	199.53 ± 53.64	0.12
	EZ	228 ± 48.80	1,537	189.33 ± 51.25		1,413	191.64 ± 52.04		1,466	193.22 ± 52.20	
HDL-C (mg/dL)	BA	50.40 ± 20.20	1,832	49.93 ± 20.75	<0.01	1,695	49.40 ± 20.35	0.05	1,420	49.88 ± 20.47	0.05
	EZ	49.90 ± 20.40	1,599	49.88 ± 20.26		1,466	48.36 ± 20.36		1,527	48.85 ± 20.24	
HbA1c (%)	BA	6.30 ± 1.31	993	6.48 ± 1.16	-0.21	1,057	6.45 ± 1.23	-0.19	919	6.43 ± 1.25	-0.10
	EZ	6.33 ± 1.42	912	6.77 ± 1.54		1,019	6.71 ± 1.54		1,061	6.51 ± 1.32	

Note: Laboratory results are presented as mean ± standard deviation (SD) because TriNetX provides only aggregated summary statistics; the number of patients contributing to each timepoint and distribution-based measures (e.g., median [IQR]) are not available. BA, bempedoic acid; EZ, Ezetimibe; SD, Standard Deviation; LDL-C, Low-Density Lipoprotein Cholesterol; HDL-C, High-Density Lipoprotein Cholesterol; HbA1c, hemoglobin A1C; SMD, standardized mean difference.

Comment 16:

Results, Page 6, Line 17: Figure 1 does not really illustrate the propensity score matching process, rather it simply shows the number of subjects excluded to create the propensity score matched groups of patients.

Response:

The authors thank the reviewer for this helpful clarification. In the revised manuscript, we have modified the figure description to accurately reflect its content.

In the revised manuscript (Page 9, Lines 5-6), the sentence now reads as follows: "Figure 1 illustrates the cohort selection process and shows the number of patients included and excluded to derive the final propensity score-matched cohorts."

Comment 17:

Results, Page 7, Section 3.2 and Table 1: use of other lipid-lowering agents by the patients, as well as other agents with potential to affect serum uric acid and gout, should be reported.

Response:

The authors thank the reviewer for this valuable suggestion. In the revised manuscript, we have added data on the baseline use of coexistent lipid-lowering agents and other medications known to influence serum uric acid levels, including statins, fibrates, PCSK9 inhibitors (evolocumab and alirocumab), niacin, diuretics, SGLT2 inhibitors, and losartan. These covariates were also incorporated into the propensity score model to account for potential confounding effects. After matching, all variables demonstrated adequate balance, with standardized mean differences (SMDs) below 0.1, confirming strong comparability between treatment groups.

In the revised manuscript (Page 10, Lines 1-9 and Table-1), the sentence now reads as follows: "In addition to baseline demographics and comorbidities, the concomitant use of lipid-modifying and UA-influencing agents was evaluated. Before matching, the use of statins (54.77% vs. 37.34%), evolocumab (2.48% vs. 11.18%), and alirocumab (0.92% vs. 4.67%) differed significantly between ezetimibe and bempedoic acid users. Following propensity score matching, the distributions of these agents were well balanced (all SMDs < 0.1). Post-matching, the frequencies of medication use were as follows: statins 37.34% vs. 37.97%, fibrates 2.91% vs. 3.39%, evolocumab 11.18% vs. 11.01%, alirocumab 4.67%

vs. 4.22%, niacin 0.55% vs. 0.59%, diuretics 23.67% vs. 22.27%, SGLT2 inhibitors 5.54% vs. 5.64%, and losartan 13.01% vs. 12.57% for bempedoic acid and ezetimibe, respectively."

Table 1. Baseline characteristics of the study cohorts before and after the matching of the propensity score.

**Characteristics**	**No. of patients in original cohort (%)**			**No. of patients after propensity score matching (%)**		
	**BA n = 7,676**	**EZ N=161,165**	**SMD**	**BA n = 7,676**	**EZ N = 7,676**	**SMD**
Age(yr), Median (IQR)	65.60 +/- 10.60	64.30 +/- 11.10	0.12	65.60 +/- 10.60	65.30 +/- 10.60	0.02
White	5487 (71.50%)	115913 (71.90%)	0.01	5487 (71.50%)	5492 (71.60%)	<0.01
Male	2624 (34.20%)	66176 (41.10%)	0.14	2624 (34.20%)	2667 (34.70%)	0.01
Asian	414 (5.40%)	5664 (3.50%)	0.09	413 (5.40%)	423 (5.50%)	<0.01
Type 1 diabetes mellitus	144 (1.90%)	3342 (2.10%)	0.01	143 (1.90%)	123 (1.60%)	0.02
Type 2 diabetes mellitus	2060 (26.80%)	51310 (31.80%)	0.11	2059 (26.80%)	1966 (25.60%)	0.03
Hypertensive disease	4987 (65.00%)	108793 (67.50%)	0.05	4986 (65.00%)	4963 (64.70%)	<0.01
Ischemia heart disease	2764 (36.00%)	56223 (34.90%)	0.02	2763 (36.00%)	2729 (35.60%)	<0.01
Cerebral infarction	290 (3.80%)	10340 (6.40%)	0.12	290 (3.80%)	278 (3.60%)	<0.01
Nontraumatic intracranial hemorrhage	25 (0.30%)	827 (0.50%)	0.03	25 (0.30%)	32 (0.40%)	0.01

Chronic kidney disease, stage 3 (Moderate)	564 (7.30%)	12986 (8.10%)	0.03	563 (7.30%)	513 (6.70%)	0.03
End stage renal disease	27 (0.40%)	1482 (0.90%)	0.07	27 (0.40%)	32 (0.40%)	0.01
Heart failure	652 (8.50%)	18405 (11.40%)	0.10	652 (8.50%)	610 (7.90%)	0.02
Occlusion and stenosis of precerebral arteries, not resulting in cerebral infarction	574 (7.50%)	10611 (6.60%)	0.04	574 (7.50%)	527 (6.90%)	0.02
Use of lipid-lower agent						
Statins	2866 (37.34%)	88270 (54.77%)	0.36	2866 (37.34%)	2914 (37.97%)	0.01
Fibrates	223 (2.91%)	5270 (3.27%)	0.02	223 (2.91%)	260 (3.39%)	0.03
Evolocumab	858 (11.18%)	3996 (2.48%)	0.36	858 (11.18%)	845 (11.01%)	<0.01
Alirocumab	358 (4.67%)	1482 (0.92%)	0.26	358 (4.67%)	323 (4.22%)	0.02
Niacin	42 (0.55%)	789 (0.49%)	<0.01	42 (0.55%)	45 (0.59%)	<0.01
Diuretics	1816 (23.67%)	45561 (28.27%)	0.11	1816 (23.67%)	1710 (22.29%)	0.04
SGLT2 inhibitors	425 (5.54%)	10105 (6.27%)	0.03	425 (5.54%)	432 (5.64%)	<0.01
Losartan	998 (13.01%)	23981 (14.88%)	0.05	998 (13.01%)	964 (12.57%)	0.01
**Laboratory**						

Cholesterol (mg/dL)	229.80 +/- 49.90	219.40 +/- 48.80	0.21	229.80 +/- 49.90	228.70 +/- 48.80	0.022
Cholesterol in LDL (mg/dL)	146.70+/- 43.10	137.90 +/- 41.30	0.21	146.70 +/- 43.10	145.50 +/- 42.20	0.029
Cholesterol in HDL (mg/dL)	49.90 +/- 20.20	47.80 +/- 19.50	0.11	49.90 +/- 20.20	49.30 +/- 20.30	0.03
C reactive protein (mg/dL)	10.40 +/- 28.80	16.30 +/- 38.70	0.17	10.40 +/- 28.80	10.50 +/- 28.20	<0.01
Hemoglobin A1C (%)	6.30 +/- 1.30	6.60 +/- 1.60	0.17	6.30 +/- 1.30	6.40 +/- 1.50	0.04
Triglyceride (mg/dL)	161.10 +/- 96.80	161.80 +/- 106.80	<0.01	161.10 +/- 96.80	160.80 +/- 93.30	<0.01
Uric acid (mg/dL)	5.40 +/- 1.20	5.70 +/- 2.00	0.19	5.40 +/- 1.20	5.30 +/- 1.50	0.07

Note: BA, bempedoic acid, and EZ, Ezetimibe, and SMD, standardized mean difference.

Comment 18:

Results, Page 8, Section 3.3.2: the second paragraph in this section that provides an interpretation of the figures for the changes in secondary outcomes over time is unnecessary. The generalizations of comparable, minimal, similar, etc., do not always align with the appearance of the figures, in part because of the scales of the graphs to include the large error bars. Suggest instead that the text simply refer to Figure 2 which shows the results for these measurements over time, and then the actual values that are reported in Table 3 can be discussed in more detail (as is done in the next paragraph).

Response:

The authors thank the reviewer for this useful comment. To improve clarity and conciseness, we have revised this section by removing the interpretive descriptions and instead directing readers to Figure 2 for visualization of longitudinal trends and to Table 3 for detailed quantitative results.

In the revised manuscript (Page 11, Lines 12-15), the sentence now reads as follows: "Figure 2 presents the longitudinal trends in serum UA, CRP, LDL-C, triglycerides, HDL-

C, and HbA1c levels over 12 months of follow-up in the bempedoic acid and ezetimibe cohorts. Corresponding mean values and standard deviations for each time point are summarized in Table 3."

Comment 19:

Results, Page 8, Lines 23-24: the sentence, "During follow-up, both bempedoic acid and ezetimibe exhibited comparable effects on lipid and metabolic parameters, although they exhibited distinct variations in specific biomarkers." appears to make 2 difference conclusions. Suggest revising for clarity.

Response:

The authors thank the reviewer for this valuable observation. We have revised the sentence for improved clarity to clearly differentiate the overall metabolic effects from the specific biomarker differences.

In the revised manuscript (Page 11, Lines 16-18), the sentence now reads as follows: "During follow-up, both bempedoic acid and ezetimibe showed generally similar effects on overall lipid and metabolic parameters overall; however, differences were observed in specific biomarkers, particularly serum UA and C-reactive protein."

Comment 20:

Results, Page 8, Lines 28-29: the statement that for total and LDL-C, "the degree of reduction was slightly more consistent in the bempedoic acid cohort" is too general and does not reflect the fact that both total and LDL-C were lower after 12 months with ezetimibe than with bempedoic acid. Also, suggest revising the similar statement in the Discussion (Page 10, Lines 14-17).

Response:

The authors thank the reviewer for this important clarification. In the revised manuscript, we have corrected the statements in both the Results and Discussion sections to accurately represent the numerical data, indicating that total cholesterol and LDL-C levels were slightly lower after 12 months in the ezetimibe cohort compared with the bempedoic acid cohort. The earlier phrasing implying greater consistency in the bempedoic acid group has been removed to ensure accuracy and clarity.

In the revised manuscript (Page 11, Lines 21-22), the sentence now reads as follows: "Although both therapies led to reductions in LDL-C and total cholesterol, the degree of reduction was slightly more consistent in the bempedoic acid cohort."

Comment 21:

Results, Page 8, Lines 29-30: suggest refining the description of the CRP results. Currently it says that CRP "decreased more markedly in patients who received bempedoic acid." According to Table 3, the baseline levels were 10.90 and 10.30 for bempedoic acid and ezetimibe, respectively, and at 12 months were 10.10 and 29.99, respectively. However, at 3 months, values with bempedoic acid were higher than with ezetimibe, and at 6 months they were relatively similar. Also, suggest revising the similar statement in the Discussion (Page 10, Lines 17-18). Please check the units reported for CRP; they are listed in Table 3 as mg/L but based on the values are likely mg/dL. Also, CRP is typically not normally distributed and should be reported as median and IQR limits instead of means and SD.

Response:

We sincerely thank the reviewer for these detailed and perceptive comments. We have revised the Results and Discussion sections to provide a more precise and balanced description of the CRP findings. The units for CRP have been verified as mg/dL and corrected throughout the manuscript. We also acknowledge that CRP levels are typically non-normally distributed; however, the TriNetX platform only provides data as mean and standard deviation values, which precluded presentation as median and interquartile range (IQR). This methodological limitation has been explicitly noted in the Discussion to ensure transparency.

In the revised manuscript section 3. Results, (Page 11, Lines 23-25), section 4. Discussion (Page 13, Lines 28-30), and section 5. Strengths and Limitations (Page 14, Lines 21-24), the sentence now reads as follows: "CRP levels, a surrogate marker of systemic inflammation, fluctuated modestly over time in both treatment groups, without a consistent pattern of difference between bempedoic acid and ezetimibe (Table 3)."

"CRP level changes were variable and did not demonstrate a consistent difference between bempedoic acid and ezetimibe in this real-world analysis, although prior clinical trials have reported modest CRP reductions with bempedoic acid."

"Fourth, because TriNetX aggregates laboratory data as means and standard deviations, we were unable to report CRP as medians with interquartile ranges, which would be more appropriate for non-normally distributed biomarkers."

Comment 22:

Results, Page 9, Line 5-13: as noted earlier, a description of the subgroup and sensitivity analyses is needed in the Methods section so that the reader can understand statements in the Results that refer generally to those subgroups, e.g., "…were largely consistent with the main findings…"

Response:

The authors thank the reviewer for this valuable suggestion. We agree that the Methods section should more clearly outline the subgroup and sensitivity analyses to aid reader comprehension. Accordingly, we have added a dedicated paragraph describing the subgroups examined and the analytical approach applied.

Specifically, subgroup analyses were conducted to evaluate the consistency of the association between bempedoic acid and hyperuricemia across key demographic and clinical characteristics.

The subgroups included:

i) Age: <65 vs. ≥65 years

ii) Sex: male vs. female

iii) Baseline uric acid levels: normal vs. elevated

iv) Metabolic comorbidities: presence of diabetes mellitus, hypertension, or chronic kidney disease

v) Use of diuretics at baseline, given their established influence on uric acid metabolism and gout risk

For each subgroup, hazard ratios (HRs) for incident hyperuricemia and initiation of anti-gout therapy were re-estimated using the same propensity score-matched cohort and Cox proportional hazards models as in the main analysis. These methodological details are now clearly described in the revised Methods section.

In the revised manuscript section 2.5 Statistical analysis, (Page 8, Lines 5-13), and section 3. Results (Page 11, Lines 30-31), the sentence now reads as follows: "To evaluate the consistency of our findings, we conducted prespecified subgroup analyses across clinically

relevant categories that could potentially modify the relationship between bempedoic acid use and hyperuricemia. Subgroups included age (<65 vs. ⩾65 years), sex, baseline UA status (normal vs. elevated), presence of diabetes mellitus, hypertension, chronic kidney disease, and baseline use of diuretics. These factors were selected based on their known associations with UA metabolism and gout risk. For each subgroup, HRs and 95% CIs for the outcomes of interest, incident hyperuricemia and initiation of anti-gout therapy, were re-estimated using Cox proportional hazards models within the propensity score-matched cohort."

"Findings from subgroups were consistent with the primary results, showing a modestly higher risk of hyperuricemia associated with bempedoic acid use across most of the subgroups."

Comment 23:

Discussion, Page 11, Lines 17-19: the point is made here that there was substantial variability in the CRP levels, which should also be recognized in the description of the Results.

Response:

The authors thank the reviewer for this insightful suggestion. We have revised both the Results and Discussion sections to consistently acknowledge the substantial variability observed in CRP levels across follow-up timepoints. The Results now indicate that CRP values fluctuated without a consistent pattern, while the Discussion highlights that this variability may reflect heterogeneous measurement timing and underlying comorbid conditions in a real-world patient population.

In the revised manuscript section 3. Results, (Page 11, Lines 23-25), and section 4. Discussion (Page 13, Lines 28-30), the sentence now reads as follows: "CRP levels, a surrogate marker of systemic inflammation, fluctuated modestly over time in both treatment groups, without a consistent pattern of difference between bempedoic acid and ezetimibe (Table 3)."

"CRP level changes were variable and did not demonstrate a consistent difference between bempedoic acid and ezetimibe in this real-world analysis, although prior clinical trials have reported modest CRP reductions with bempedoic acid."

Comment 24:

Discussion: While the authors have briefly mentioned some limitations of their analyses, a stronger acknowledgement of these limitations is needed. Suggest recognizing the possibility for a type I error (longer follow-up may have demonstrated a significant effect). Also, as mentioned earlier in the comments, suggest carefully comparing these results to those from CLEAR Outcomes, including a direct comparison of the magnitude of the incidence for hyperuricemia and gout, and considering differences in the way these were defined. Furthermore, the likelihood that some patients with mild gout symptoms may have received anti-inflammatory and/or analgesic drugs for their symptoms does not appear to be captured by this analysis that examined use of “anti-gout medications.” Thus, the true incidence of symptomatic gout has likely been underestimated. Given the shorter period of follow-up compared to CLEAR Outcomes, could the lack of association between bempedoic acid use and incident gout, despite greater incidence of hyperuricemia, be simply due to a type II statistical error?

Response:

The authors thank the reviewer for these perceptive comments. We have strengthened the Discussion to more thoroughly acknowledge potential limitations, including the possibilities of both type I and type II errors.

Regarding type I error, the observed association between bempedoic acid and elevated serum uric acid is biologically plausible, given its inhibition of renal OAT transporters, and is consistent with prior clinical evidence, including findings from the CLEAR Outcomes trial.

Regarding type II error related to incident gout, our analysis focused on the initiation of anti-gout medications, which reflects pharmacologic treatment for clinically significant gout in real-world practice. We recognize that milder gout episodes managed with over-the-counter anti-inflammatory or analgesic agents may not have been captured, potentially underestimating the true incidence of symptomatic gout. However, because an active comparator design was used, this limitation is expected to occur similarly in both treatment groups. We have clarified these points in the manuscript, including a direct comparison with CLEAR outcomes regarding the magnitude of hyperuricemia and gout incidence, and explicitly discussed the potential influence of both type I and type II errors on our findings.

In the revised manuscript Section 4. Discussion (Page 12, Lines 17-31 and Page 13, Lines 1-5), and section 5. Strengths and Limitations (Page 14, Lines 24-28), the sentence now reads as follows: "In the CLEAR Outcomes trial, bempedoic acid was associated with modest increases in serum UA and a slightly higher incidence of gout (3.1% vs. 2.1%), consistent with the mechanism of inhibition of renal OAT2. When compared with the CLEAR Outcomes trial, our real-world analysis demonstrated a similar relative increase in hyperuricemia, but no corresponding increase in gout treatment initiation. This discrepancy likely reflects differences in event ascertainment, our study defined clinically significant hyperuricemia based on recorded UA levels and the prescription of anti-gout medications, while CLEAR outcomes relied on adjudicated clinical events. It is also plausible that patients with mild or self-limited gout flares managed with non-specific anti-inflammatory or analgesic agents were not captured in our dataset, resulting in an underestimation of symptomatic gout. Our findings complement those of the CLEAR Outcomes program and its recent sub analysis by Ray et al., who reported that the concomitant use of urate-lowering therapies may attenuate the risk of gout in patients receiving bempedoic acid.[22] This reinforces the clinical importance of monitoring UA levels and considering preventive management in high-risk individuals. Furthermore, our median follow-up duration was shorter than that of CLEAR results (approximately 12 vs. 40 months), which may have limited statistical power to detect small differences in incident gout. Therefore, the absence of an association between bempedoic acid use and gout should be interpreted cautiously, as a Type II error cannot be excluded. Conversely, multiple secondary comparisons may increase the potential for Type I error, further emphasizing the need for replication in longer-term prospective studies."

"Fifth, given the observational design and limited follow-up duration, the potential for both Type I and Type II statistical errors exists. However, the observed association is supported by well-established biological mechanisms: bempedoic acid increases serum UA by inhibiting of renal OAT transporters and the direction of effect is consistent with prior clinical evidence, including findings from CLEAR outcomes."

Comment 25:

Conclusions, Page 12, Line 1: instead of indicating that routine UA surveillance or intervention "is not acceptable" suggest writing it as "may not be necessary."

Response:

The authors thank the reviewer for this thoughtful suggestion. We agree that the phrase "may not be necessary" more accurately reflects the observational nature of our study. Accordingly, the conclusion has been revised to reflect this more measured interpretation.

In the revised manuscript Page 15, Lines 26-28, the sentence now reads as follows: "Although clinicians should be aware of this possible metabolic side effect, routine UA surveillance or intervention may not be necessary for most patients, except those with known risk factors for gout or renal failure."

Comment 26:

Figure 2: units for the laboratory variables are missing from the figure; also, include the number of patients.

Response:

The authors thank the reviewer for this helpful suggestion. In the revised manuscript, Figure 2 has been updated to include the appropriate units for all laboratory variables. The revised figure is presented on Page 19, Figure 2.

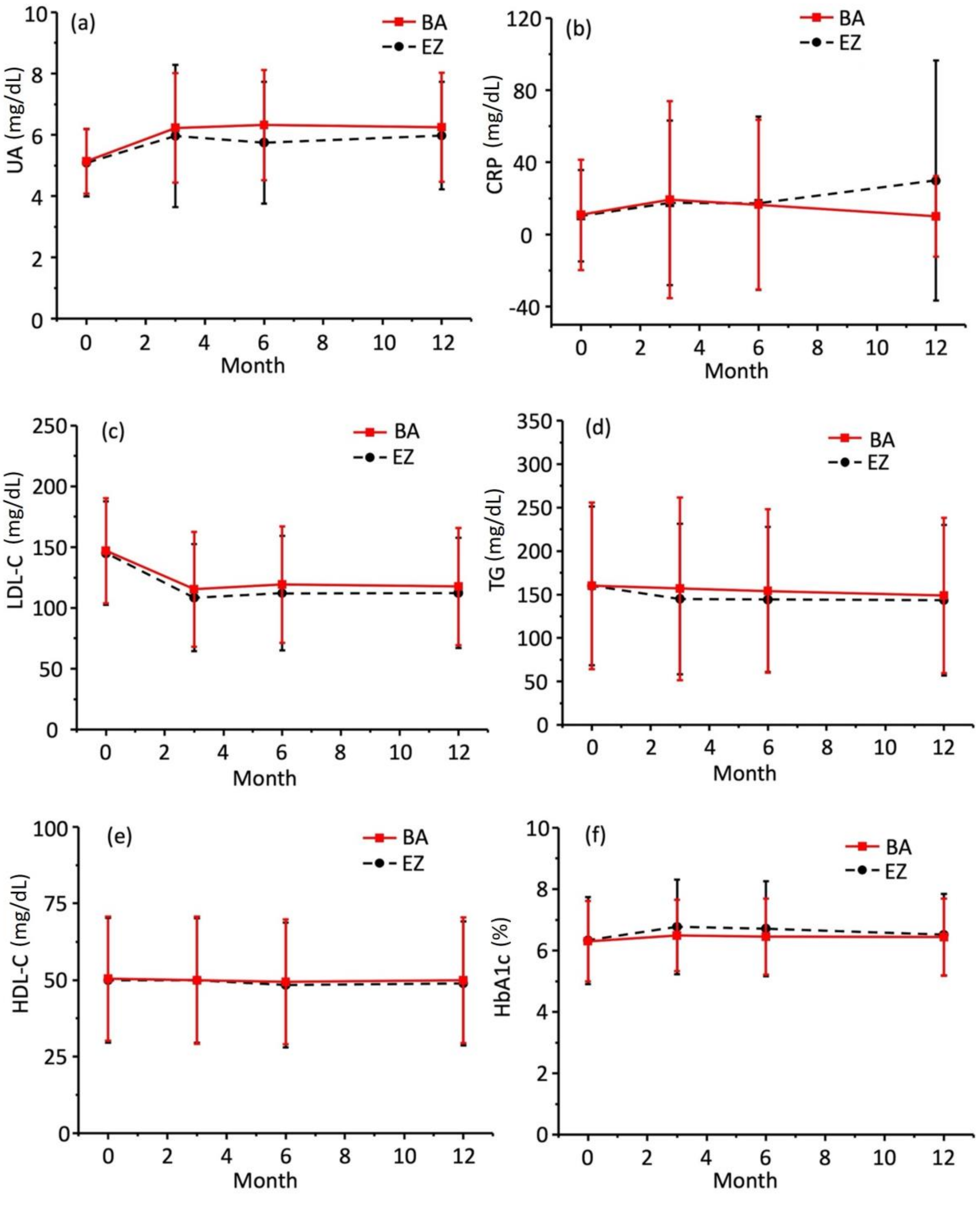

Comment 27:

Table 3: the number of patients in each analysis are missing; also, triglycerides and CRP are not usually normally distributed and should be reported as medians and IQR limits instead of means and SDs.

Response:

The authors thank the reviewer for this important observation. We agree that reporting the number of patients per timepoint and presenting triglycerides and CRP as medians with interquartile ranges (IQR) would be ideal. However, due to the structure of the TriNetX research platform, laboratory results are available only as aggregated summary statistics (mean ± SD), and the number of patients contributing at each timepoint is not accessible. Accordingly, we have clarified these data limitations in both the Methods section and the footnote of Table 3.

In the revised manuscript section 2.Methods (Page 8, Lines 20-23) and the table-3 footnote (Page 27, Lines 1-2), the sentence now reads as follows: "Laboratory results were retrieved from the TriNetX platform, which provides aggregated summary statistics (mean ± standard deviation) for each time point but does not disclose the number of patients who contributing. Consequently, laboratory parameters are presented as mean ± SD without sample size data."

"**Note:** Laboratory results are presented as mean ± standard deviation (SD) because TriNetX provides only aggregated summary statistics; the number of patients contributing to each timepoint and distribution-based measures (e.g., median [IQR]) are not available."

Minor comments:

Comment 1:

Abstract: suggest defining abbreviations at their first use (unless the journal allows use of certain abbreviations without definitions)

Response:

The authors thank the reviewer for this helpful reminder. In the revised manuscript, all abbreviations have been defined at first mention in the abstract to ensure clarity and consistency with journal style requirements.

In the revised manuscript, the abstract (page 2, lines 2-18) now reads as follows: "Bempedoic acid, an adenosine triphosphate citrate lyase inhibitor, is approved to lower low-density lipoprotein cholesterol (LDL-C) cholesterol in patients with hyperlipidemia requiring additional reduction in lipids. However, its effects on serum uric acid (UA) are not yet fully defined, with potential implications for the risk of gout in clinical practice. We

examined whether bempedoic acid increases hyperuricemia compared to ezetimibe in patients with hyperlipidemia using a multi-institutional electronic health records database. Using propensity score matching between treatment groups, 7,676 patients receiving bempedoic acid were matched to 7,676 patients receiving ezetimibe. The incidence of hyperuricemia was significantly higher in the bempedoic acid group (Hazard ratio (HR): 1.94; 95% confidence interval (CI): 1.58 - 2.37; $p < 0.01$) compared to the ezetimibe group. However, no significant differences were observed in the initiation of anti-gout therapy (HR: 1.06; 95% CI: 0.86 - 1.29; $p = 0.59$). Changes in serum UA and other metabolic markers, such as LDL-C, high-density lipoprotein cholesterol, triglycerides, total cholesterol, C-reactive protein, and glycated hemoglobin, were similar between groups. These findings suggest that while bempedoic acid may elevate serum UA, its clinical impact on gout treatment needs appears limited, supporting its continued use in patients requiring further LDL-C lowering while emphasizing the need for monitoring in individuals at risk of hyperuricemia."

Comment 2:

Throughout the Methods, analyzes should be spelled analyses.

Response:

The authors thank the reviewer for this helpful observation. In the revised manuscript, all instances of "analyzes" have been corrected to "analyses".

Comment 3:

Methods, Page 5, Line 27 and Discussion, Page 11, Line 8: suggest replacing "confusion" with "confounding."

Response:

The authors thank the reviewer for this valuable comment. In the revised manuscript, all instances of "confusion" have been corrected to "confounding".

Comment 4:

Discussion, Page 10, Line 25: suggest revising to "Our real-world data support the findings of the clinical trials…"

Response:

The authors thank the reviewer for this valuable suggestion. In the revised manuscript (Page 12, Line 16), the sentence has been updated to read: "Our real-world data support the findings of the clinical trials…"

Comment 5:

Table 1: please include the units for the laboratory values; please use Arabic numerals for diabetes, ie, Type 1 and Type 2 diabetes mellitus.

Response:

The authors thank the reviewer for this valuable suggestion. In the revised manuscript, Table 1 has been updated to include units for all laboratory values and diabetes types have been converted to Arabic numerals (Type 1 and Type 2 diabetes mellitus) for clarity and consistency.

Reviewer2:

We sincerely thank the reviewer for the thoughtful and constructive comments. Each point has been carefully addressed below, and the manuscript has been revised accordingly to enhance clarity, accuracy, and transparency.

Major comments:

Comment 1:

1) General:

a) Bempedoic acid is also available as a combination tablet with ezetimibe. Did you exclude patients on combination use?

Response:

The authors thank the reviewer for this comment. We agree that combination therapy with ezetimibe is an important consideration. We have considered this in our analyses. During 12 months of follow-up, Cox proportional hazards modeling demonstrated that bempedoic acid use was associated with a markedly increased risk of developing hyperuricemia (HR: 2.352; 95% CI: 1.605–3.446; $p < 0.001$).

Although the risk of hyperuricemia was elevated, the initiation of anti-gout therapy did not differ significantly between the groups. Specifically, the adjusted risk of starting antigout therapy in the bempedoic acid group was HR: 1.131; 95% CI: 0.809–1.582; $p = 0.390$.

The results remained consistent, showing an increased risk of uric acid elevation, with no statistically significant differences observed in the use of allopurinol, febuxostat, or colchicine between groups.

b) Include the manuscript Ray et al, within the Discussion section that was recently published in JACC: advances which focuses on use of uric acid lowering medications in CLEAR Outcomes trial.

Response:

We appreciate this valuable suggestion and have now cited the recent study by Ray KK et al. (JACC: Advances, 2025) in the Discussion section to strengthen the clinical context. This study extends the insights of the CLEAR Outcomes trial by examining the potential mitigating role of urate-lowering therapies in patients treated with bempedoic acid.

In the revised manuscript section 4. Discussion (Page 12, lines 27-30), the sentence now reads as follows: "Our findings complement those of the CLEAR Outcomes program and its recent sub analysis by Ray et al., who reported that the concomitant use of urate-lowering therapies may attenuate the risk of gout in patients receiving bempedoic acid.[22] This reinforces the clinical importance of monitoring UA levels and considering preventive management in high-risk individuals."

Reference:

22. Ray KK, Nicholls SJ, Lincoff AM, Li N, Powell HA, Herout PM, Bloedon L, Nissen SE. Association of Uric Acid-Lowering Therapies on Gout Frequency with Bempedoic Acid: Clinical Insights From CLEAR Outcomes. JACC Adv. 2025 Sep 26;4(11 Pt 1):102207.

c) Did you look at gout in over the index period? If not, why not since the risk of elevated serum uric acid is gout.

Response:

We agree with the reviewer that the risk of gout is a clinically relevant downstream consequence of hyperuricemia. In this study, we evaluated the new initiation of anti-gout medications (including allopurinol, febuxostat, and colchicine) as a proxy for clinically significant gout, as the direct diagnosis coding of gout episodes was

incomplete in all data sources within TriNetX. This approach allowed us to capture the initiation of gout-related treatment in real-world practice. We have clarified this methodology in the Methods section.

In the revised manuscript (Page 6, line 31 and Page 7, lines 1-2), the sentence now reads as follows: "Because the diagnostic coding for gout is inconsistently available in TriNetX, the initiation of anti-gout medications (e.g., allopurinol, febuxostat, and colchicine) was used as a validated proxy for incident gout events."

Comment 2:

Page 4, lines 18-20: Please confirm that this study was conducted using the global TriNetX network (the true "federated global network") or the more commonly used US TriNetx dataset. The n's and demographics are more representative of the US dataset.

Response:

We appreciate the careful observation of the reviewer. This study was conducted using the TriNetX US Collaborative Network. The US dataset was selected to ensure consistency in clinical coding standards (ICD-10-CM and LOINC), completeness of laboratory data, and population representativeness for US clinical practice. Accordingly, the overall sample size and demographic composition align with the US network.

In the revised manuscript (Page 5, lines 19-21), the sentence now reads as follows: "The retrospective cohort study used data from the TriNetX U.S. Collaborative Network, a federated research platform comprising electronic medical records from approximately 131 million patients across 71 healthcare organizations."

Comment 3:

Page 4, lines 29-30: Conceptually, not sure this makes sense. You can't mimic a RCT with a retrospective observational database study, as there is no intervention. I think what they mean is that they had pre- and post-index observation periods. It is misleading to say that they mimicked an RCT. Perhaps they are referring to the propensity score matching process? but that only is trying to mimic the randomization aspect of an RCT.

Response:

The authors thank the reviewer for this important clarification. We have revised the text to state that the study followed a structured cohort design with defined index, baseline, and follow-up periods and used propensity score matching to approximate balance between

treatment groups, thus addressing difficult and improving comparability without implying that the study mimics a randomized controlled trial.

In the revised manuscript (Page 5, lines 29-31 and Page 6, lines 1-4), the sentence now reads as follows: "This retrospective cohort study was structured to resemble a randomized comparison, with clearly defined index, baseline, and follow-up periods. The index date was defined as the first recorded prescription for study medications. Baseline characteristics were assessed using data prior to the index date. Adult patients (aged ≥18 years) with a diagnosis of hyperlipidemia who initiated bempedoic acid or ezetimibe were eligible. To isolate incident cases of hyperuricemia, individuals with a prior diagnosis of hyperuricemia or the use of anti-gout therapy before the index date were excluded."

Comment 4:

Page 5, lines 9-11: What were the years of index rx identification / data collection?

- What was the pre-index observation period? 12 mos pre? I believe it said 12 mos post
- How were patients determined to be "New users" of bempedoic acid or ezetimibe (related to duration of the pre-index observation period)
- What about baseline and pre-index rx utilization?

Response:

We thank the reviewer for these important questions.

i) The study period extended from January 1, 2020, to May 3, 2025, corresponding to the time during which bempedoic acid was available in the TriNetX US Collaborative Network.

ii) To identify new users, patients were required to have no prescription record for the index medication (bempedoic acid or ezetimibe) during the 3 years preceding the index date (washout period).

iii) Consistent with standard pharmacoepidemiologic methods, a 12-month pre-index (lookback) period was applied to assess baseline comorbidities, concomitant medications, and other covariates. This approach ensured sufficient capture of the pre-index clinical context and reduced potential misclassification bias.

iv) The post-index observation period extended for 12 months of follow-up or until the first day of discontinuation of medication, death, or end of data availability.

In the revised manuscript (Page 6, Lines 14-19), the sentence now reads as follows: "The study period extended from January 1, 2020, to May 3, 2025, corresponding to the period of bempedoic acid availability in the TriNetX U.S. Collaborative Network. To identify new users, patients were required to have no prescription record for index medication during the year prior to index date (washout period). A 12-month look-back period before the index date was applied to assess baseline comorbidities, concomitant medications, and other covariates. Follow-up continued for post-index or until death, treatment discontinuation, or the end of data availability."

Comment 5:

Page 6, lines 8-10: What was the match? 1:1, 1:2? how were patients matched?

What were the 2 groups matched on? all of the baseline demographic and clinical characteristics? only select ones?

Response:

The authors thank the reviewer for this comment. The patients were matched using a 1:1 propensity score matching approach. The propensity score was estimated using a logistic regression model that incorporates all available baseline demographic and clinical characteristics, including age, sex, comorbidities, and concomitant medications, as listed in Table 1. The match was performed using a replacement nearest-neighbor algorithm, with a caliper of 0.1 standard deviations from the logit of the propensity score to ensure close comparability between the two treatment groups.

Comment 6:

Page 5, lines 2-5: was past medical history of gout excluded?

Response:

We appreciate the insightful question of the reviewer. Yes, to ensure that only incident hyperuricemia or gout-related events were captured, patients with a documented history of gout (ICD-10-CM codes M10.x) before the index date were excluded from the cohort. This exclusion was in addition to removing individuals with pre-existing hyperuricemia or prior use of antigout therapy. We have now clarified this explicitly in the Methods section.

In the revised manuscript (Page 6, Lines 11-13), the sentence now reads as follows: "To isolate incident cases of hyperuricemia and gout, patients with a documented history of

gout, a prior diagnosis of hyperuricemia, or any use of anti-gout therapy before the index date were excluded."

Minor comments:

Comment 7:

The manuscript needs to be reviewed carefully for grammatical edits. Examples just in the abstract include page 2, lines 2-3; line 7.

Response:

The authors thank the reviewer for this comment. The revised manuscript has been carefully reviewed and corrected for grammatical accuracy and clarity, including the Abstract and all other sections.

Comment 8:

Page 3, Lines 10-13: bempedoic acid is approved and the indication referenced should be revised to be current (it is indicated beyond ASCVD or HeFH now)

Response:

The authors thank the reviewer for this important clarification. The description of the approved indication of bempedoic acid has been updated to reflect the most recent US FDA label, which now includes adults with primary hyperlipidemia, with or without established ASCVD or HeFH.

In the revised manuscript (Page 4, Lines 10-13), the sentence now reads as follows: "Bempedoic acid, an oral adenosine triphosphate-citrate lyase inhibitor, is approved for use in adults with primary hyperlipidemia, including those with or without atherosclerotic cardiovascular disease (ASCVD) or heterozygous familial hypercholesterolemia (HeFH) who require additional lowering of low-density lipoprotein cholesterol (LDL-C)[7]."

Reference:

7. Mach F, Koskinas KC, Roeters van Lennep JE, Tokgözoğlu L, Badimon L, Baigent C, Benn M, Binder CJ, Catapano AL, De Backer GG, Delgado V, Fabin N, Ference BA, Graham IM, Landmesser U, Laufs U, Mihaylova B, Nordestgaard BG, Richter DJ, Sabatine MS; ESC/EAS Scientific Document Group. 2025 Focused Update of the 2019 ESC/EAS Guidelines for the management of dyslipidaemias. Eur Heart J. 2025 Nov 7;46(42):4359-4378.

Comment 9:

Page 3, lines 23-24: clarify that what is not understood is any implications to increased serum uric acid in the real-world setting. Check for this clarification throughout manuscript.

Response:

The authors are grateful for the suggestion of the reviewer. We have clarified that the key uncertainty relates to the clinical implications of increases associated with bempedoic acid in serum uric acid in real-world practice. The text has been revised accordingly in the revised manuscript and we ensured consistent phrasing throughout the manuscript to emphasize that while the biochemical effect is established, its real-world clinical relevance remains less well defined.

In the revised manuscript (Page 5, Lines 7-9), the sentence now reads as follows: "Although bempedoic acid is known to increase serum UA levels, the clinical implications of this effect in real-world settings remain uncertain."

Comment 10:

Page 4, lines 11-15: Remove results from the introduction section.

Response:

The authors thank the reviewer for this important observation. We agree that the original text included an early summary of the study findings, which is more appropriate for the results or discussion sections. Consequently, the introduction has been revised to focus solely on the rationale and objectives of the study.

Comment 11:

Page 6, line 2: uric acid is not an inflammatory marker and should be updated.

Response:

The authors thank the reviewer for this important clarification. Uric acid is not classified primarily as an inflammatory marker. Consequently, the manuscript has been revised to accurately describe the role of uric acid in the study, highlighting its association with cardiovascular risk factors and hyperuricemia, rather than presenting it solely as an inflammatory marker.

In the revised manuscript (Page 7, Lines 19-21), the sentence now reads as follows: "Uric acid levels were also incorporated; although UA is not a classical inflammatory marker, it has been associated with cardiovascular risk and metabolic disturbances."

Comment 12:

Page 6, lines 12-14: exploratory analyses referenced but not included- please address.

Response:

The authors thank the reviewer for noting this and appreciate the careful review. Reference to "exploratory analyses" in the Methods section was accidentally included and does not correspond to any analyzes performed in this study. No exploratory analyzes were performed beyond the prespecified primary and subgroup analyzes.

To correct this, the sentence-referencing exploratory analyses has been removed from the revised manuscript, ensuring consistency and accuracy between the Methods and Results sections. The authors thank the reviewer for highlighting this, which allowed us to clarify and streamline the description of our analytic approach.

In the revised manuscript (Page 8, Lines 23-24), the sentence now reads as follows: "All analyses were performed within the TriNetX analytics platform and were prespecified based on clinically relevant hypotheses and prior evidence."

Comment 13:

Page 8, line 5: describe how anti-gout medications were defined.

Response:

The authors thank the reviewer for this important observation. In the revised manuscript (Page 6, Line 31 and Page 7, Lines 1-2), we have clarified that antigout medications were defined as "Because the diagnostic coding for gout is inconsistently available in TriNetX, the initiation of anti-gout medications (e.g., allopurinol, febuxostat, and colchicine) was used as a validated proxy for incident gout events." This definition has been added to the Methods section for clarity.

Comment 14:

Page 9, lines 27-28: incidence of gout numbers from CLEAR Outcomes is incorrect. Data is 3.1% vs 2.1%

Response:

The authors thank the reviewer for this important observation. The revised manuscript (Page 12, Lines 18-20) has been corrected to reflect the accurate incidence of gout reported " In the CLEAR Outcomes trial, bempedoic acid was associated with modest increases in serum UA and a slightly higher incidence of gout (3.1% vs. 2.1%), consistent with the mechanism of inhibition of renal OAT2." This correction has been applied discussion sections to ensure accuracy and consistency.

Comment 15:

Figure 2 for CRP. ZE should be EZ. Also, need to add n's at the time points so you know how many patients data is available.

Response:

The authors thank the reviewer for this important observation. Figure 2 has been revised to correct the label from ZE to EZ and to include the number of patients (n) at each time point for all laboratory variables, providing transparency regarding the availability of data.

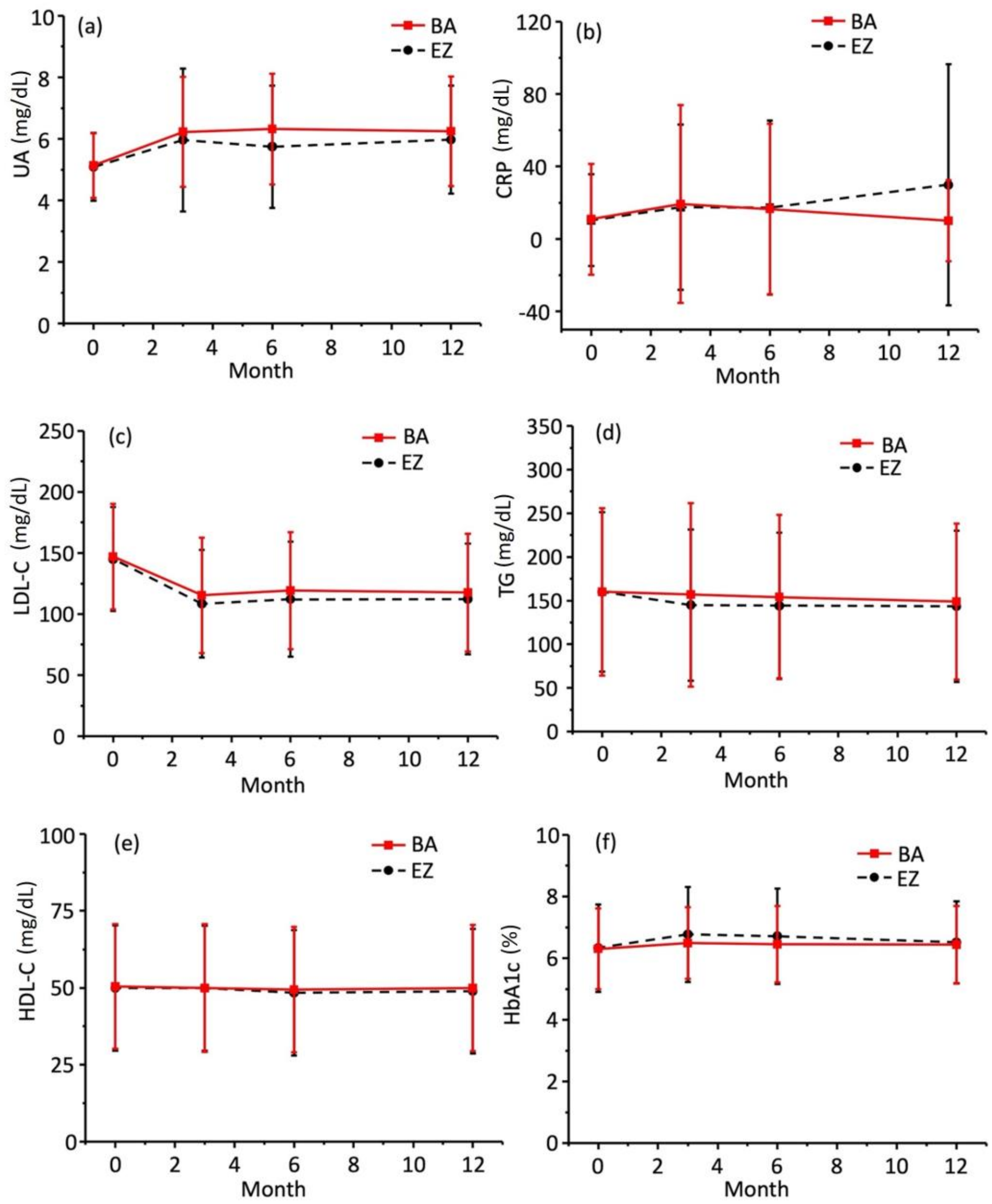

Comment 16:

Table 3: add n's

Response:

The authors thank the reviewer for this important suggestion. In the revised manuscript, Table 3 has been updated to include the number of patients (n) who contribute to each laboratory measurement at the time points 3, 6, and 12 months. This provides transparency on the sample size for each analysis and allows readers to interpret the results more accurately.

Table 3. Longitudinal changes in laboratory biomarkers at baseline, 3, 6, and 12 months.

Laboratory Parameter	Drug	Baseline (Mean ± SD)	3 months numbers	3 Months (Mean ± SD)	SMD	6 months numbers	6 Months (Mean ± SD)	SMD	12 months numbers	12 Months (Mean ± SD)	SMD
Uric Acid (mg/dL)	BA	5.13 ± 1.05	171	6.22 ± 1.78	0.13	115	6.32 ± 1.80	0.30	116	6.24 ± 1.78	0.16
	EZ	5.09 ± 1.10	66	5.96 ± 2.32		54	5.74 ± 1.98		60	5.97 ± 1.75	
C-Reactive Protein (mg/dL)	BA	10.90 ± 30.60	127	19.29 ± 54.53	0.03	113	16.49 ± 47.02	-0.02	114	10.10 ± 22.34	-0.40
	EZ	10.30 ± 25.30	126	17.58 ± 45.57		133	17.33 ± 48.03		105	29.99 ± 66.52	
LDL-C (mg/dL)	BA	147 ± 43.10	1,823	115.37 ± 47.21	0.15	1,680	119.21 ± 47.89	0.15	1,412	117.70 ± 48.24	0.12
	EZ	145 ± 42.50	1,590	108.46 ± 44.15		1,454	112.22 ± 46.92		1,513	112.28 ± 45.22	
Triglycerides (mg/dL)	BA	160 ± 95.80	1,818	156.80 ± 105.09	0.12	1,689	154.06 ± 94.15	0.11	1,417	148.91 ± 89.55	0.06
	EZ	160 ± 91.30	1,600	144.94 ± 86.48		1,466	144.22 ± 83.41		1,523	143.44 ± 86.53	

Total Cholesterol (mg/dL)	BA	230 ± 49.80	1,743	198.01 ± 53.50	0.17	1,608	201.00 ± 53.45	0.18	1,376	199.53 ± 53.64	0.12
	EZ	228 ± 48.80	1,537	189.33 ± 51.25		1,413	191.64 ± 52.04		1,466	193.22 ± 52.20	
HDL-C (mg/dL)	BA	50.40 ± 20.20	1,832	49.93 ± 20.75	<0.01	1,695	49.40 ± 20.35	0.05	1,420	49.88 ± 20.47	0.05
	EZ	49.90 ± 20.40	1,599	49.88 ± 20.26		1,466	48.36 ± 20.36		1,527	48.85 ± 20.24	
HbA1c (%)	BA	6.30 ± 1.31	993	6.48 ± 1.16	-0.21	1,057	6.45 ± 1.23	-0.19	919	6.43 ± 1.25	-0.10
	EZ	6.33 ± 1.42	912	6.77 ± 1.54		1,019	6.71 ± 1.54		1,061	6.51 ± 1.32	

Note: Laboratory results are presented as mean ± standard deviation (SD) because TriNetX provides only aggregated summary statistics; the number of patients contributing to each timepoint and distribution-based measures (e.g., median [IQR]) are not available. BA, bempedoic acid; EZ, Ezetimibe; SD, Standard Deviation; LDL-C, Low-Density Lipoprotein Cholesterol; HDL-C, High-Density Lipoprotein Cholesterol; HbA1c, hemoglobin A1C; SMD, standardized mean difference

Comment 17:

Page 18, for the laboratory values, need to add units. Use LDL-C vs "Cholesterol in LDL"- same for HDL.

Response:

The authors thank the reviewer for this important observation. The laboratory values in the manuscript and the figures have been updated to include units, and the terminology has been revised for clarity, using LDL-C and HDL-C consistently throughout the text, tables, and figures.

Response to Reviewers Comments

We sincerely thank the reviewers for their thoughtful, detailed, and constructive comments, which have substantially improved the clarity, accuracy, and rigor of this manuscript. We have carefully addressed each point raised and revised the manuscript, accordingly, including clarifying definitions of biochemical and clinically significant hyperuricemia, explicitly reporting discordant cases, refining language to avoid equivocal interpretations, addressing the use of combination bempedoic acid/ezetimibe therapy through additional analyses, and transparently describing the limitations inherent to the TriNetX platform with respect to laboratory data reporting.

We have also revised the Abstract, Plain Language Summary, Introduction, Methods, Results, Discussion, and Strengths and Limitations sections to ensure that all conclusions are directly supported by the data and accurately reflect both existing clinical trial evidence and real-world clinical practice. We believe these revisions have strengthened the manuscript and improved its interpretability and relevance for clinicians and researchers.

We appreciate the opportunity to revise our work and are grateful to the reviewers and editors for their time and expertise.

Reviewer1:

The authors sincerely thank the reviewer for the thoughtful and constructive comments. Each point has been carefully addressed below, and the manuscript has been revised accordingly to improve clarity, accuracy, and transparency.

Comment 1:

To address the previous request to include information about patients who were discordant (i.e., had elevated uric acid but no use of anti-gout therapy OR had use of anti-gout therapy but uric acid was not elevated), the authors added to the Methods section that discordant cases were analyzed separately. However the results of this discordant analysis do not appear to have been incorporated into the Results section of the manuscript.

Response:

We thank the reviewer for this careful follow-up and for highlighting the need to explicitly present the results of the discordant analyses. In response, we have now incorporated the results of the discordant cases directly into the Results section, in addition

to their description in the Methods. Specifically, we report the number of patients who developed elevated serum uric acid without subsequent initiation of anti-gout therapy, as well as those who initiated anti-gout therapy without documented hyperuricemia, in each treatment group.

Revised text (page 10, line 29 to page 11, line 4)

In addition to the primary outcome analyses, discordant outcome patterns were examined. Among patients who developed biochemical hyperuricemia (serum uric acid >7.0 mg/dL) without subsequent initiation of anti-gout therapy, 185 patients (2.41%) were in the bempedoic acid group and 86 patients (1.12%) were in the ezetimibe group. Conversely, among patients who initiated anti-gout therapy without a documented elevation in serum uric acid, 134 patients (1.75%) were in the bempedoic acid group and 121 patients (1.58%) were in the ezetimibe group. These discordant cases were analyzed separately and did not materially alter the primary findings.

Comment 2:

Previously, it was suggested that the authors clarify the following statement about total and LDL-C responses: "the degree of reduction was slightly more consistent in the bempedoic acid cohort." While the authors indicate in their rebuttal that they "corrected these statements in the Results and Discussion sections to accurately represent the numerical data, indicating that total cholesterol and LDL-C levels were slightly lower after 12 months in the ezetimibe cohort compared with the bempedoic acid cohort" this does not seem to be the case. On Page 11, Lines 304-305 of the Results section still state, "Although both therapies led to reductions in LDL-C and total cholesterol, the degree of reduction was slightly more consistent in the bempedoic acid cohort." Similarly, Page 13, Lines 371-372 state, "both bempedoic acid and ezetimibe were similarly effective in lowering LDL-C and total cholesterol, and the bempedoic acid cohort showed slightly more consistent reductions."

Response:

We thank the reviewer for carefully re-evaluating the revised manuscript and for pointing out the remaining inconsistency in the Results and Discussion sections. In response, we have now removed all qualitative comparative language suggesting differential consistency or magnitude of lipid lowering between treatment groups. The

Results and Discussion sections have been revised to state that both bempedoic acid and ezetimibe were associated with similar reductions in LDL-C and total cholesterol over 12 months, without implying superiority or greater consistency of effect for either agent.

This revision ensures that the manuscript accurately reflects the descriptive nature of the laboratory analyses and avoids interpretive language in the absence of formal statistical comparisons.

Revised text:

Results section (Page 11, Lines 24-26)

Although both therapies led to reductions in LDL-C and total cholesterol over the 12-month follow-up period, the overall patterns of lipid lowering were similar between the bempedoic acid and ezetimibe cohorts.

Discussion section (Page 13, Lines 29-30)

Both bempedoic acid and ezetimibe were associated with comparable reductions in LDL-C and total cholesterol over the 12-month follow-up period.

Comment 3:

Another reviewer had commented that bempedoic acid is also available as a combination tablet with ezetimibe and asked if subjects using the combination were excluded. In their response to this comment the authors provide hazard ratios for developing hyperuricemia and for use of antigout therapy “in the bempedoic acid group” but it isn’t clear how this response addresses the comment regarding a combination bempedoic acid/ezetimibe tablet.

Response:

We thank the reviewer for this important clarification and apologize that our previous response did not explicitly address the handling of the fixed-dose bempedoic acid/ezetimibe combination tablet.

In the primary analysis, patients receiving the fixed-dose combination of bempedoic acid and ezetimibe were not excluded. Exposure groups were defined based on the index medication initiated, allowing combination therapy during follow-up in both cohorts, consistent with real-world clinical practice.

To address the reviewer’s concern regarding potential exposure misclassification, we additionally conducted a sensitivity analysis excluding all patients with any record of fixed-

dose bempedoic acid/ezetimibe combination therapy. The results of this restricted analysis were consistent with the primary findings, demonstrating an increased risk of biochemical hyperuricemia associated with bempedoic acid use and no significant difference in the initiation of anti-gout therapy.

We have now clarified the handling of combination therapy and the results of this sensitivity analysis in the Methods and Results sections to avoid ambiguity.

Revised text:

In method section (page 8, line 8-11)

In the primary analysis, exposure groups were defined by the index medication initiated; patients receiving the fixed-dose bempedoic acid/ezetimibe combination were not excluded. A sensitivity analysis was additionally performed excluding all patients with any record of fixed-dose combination therapy.

In result section (page 11, line 5-9)

In a sensitivity analysis excluding patients who received the fixed-dose bempedoic acid/ezetimibe combination tablet, the association between bempedoic acid use and incident hyperuricemia remained significant (HR: 2.35; 95% CI: 1.61–3.45; $p < 0.01$), and no difference was observed in the initiation of anti-gout therapy between groups (HR: 1.13; 95% CI: 0.81–1.58; $p = 0.39$).

Comment 4:

The authors have explained in their responses and in the manuscript that they could not provide the numbers of subjects included for the secondary outcomes because the TriNetX analytics platform provides aggregated summary statistics for each time point but does not disclose the number of patients who contributed. However, in some of their responses, the authors indicate that they have updated the tables and figures to include “the number of patients (n) at each time point for all laboratory variables, providing transparency regarding the availability of data.” Please check throughout the manuscript and clarify.

Response:

We thank the reviewer for noting this apparent inconsistency and appreciate the opportunity to clarify.

In the TriNetX analytics platform, laboratory results are provided as aggregated summary statistics. While Table 3 reports the number of patients with available laboratory data within each follow-up time window, the platform does not disclose analyte-specific sample sizes for individual laboratory variables (e.g., serum uric acid, LDL-C, CRP) at each time point.

Accordingly, the numbers reported in Table 3 reflect the overall number of patients with at least one laboratory measurement available during the corresponding time window, rather than the exact number contributing to each individual laboratory parameter. We have now clarified this distinction in the Methods section and the Table 3 footnote to ensure transparency and consistency throughout the manuscript.

Revised text:

In method (page 8, line 17-21)

For secondary laboratory outcomes, the TriNetX platform provides aggregated summary statistics. The reported numbers of patients at each follow-up time point reflect the total number of patients with at least one laboratory record available within that time window and do not represent analyte-specific sample sizes.

In footnote (page 26, line 2-3)

n indicates the number of patients with at least one laboratory measurement available within the specified follow-up time window. Analyte-specific sample sizes are not available in the TriNetX platform.

Reviewer2:

We sincerely thank the reviewer for the thoughtful and constructive comments. Each point has been carefully addressed below, and the manuscript has been revised accordingly to enhance clarity, accuracy, and transparency.

Comment 1:

Abstract, Lines 4-5: “However, its effects on serum uric acid (UA) are not yet fully defined, with potential implications for the risk of gout in clinical practice.” The effects of bempedoic acid on serum uric acid have been studied in 4 phase 3

randomized controlled trials, and one phase 3 cardiovascular outcomes trial which has consistently demonstrated BA increases serum uric acid by 0.8 mg/dL compared to placebo. What you are assessing are the effects of bempedoic acid on serum acid with real world use. Thus, you should appropriately reword this sentence to something like "However data regarding the effects of bempedoic acid on serum uric acid levels and the use of anti-gout therapy with real world use is lacking."

Response:

We thank the reviewer for this important clarification. We agree that the effects of bempedoic acid on serum uric acid have been consistently demonstrated in phase 3 randomized controlled trials and in a cardiovascular outcomes trial. Our intent was to highlight the lack of data regarding real-world use, rather than the absence of randomized evidence.

We have revised the abstract to clarify that while increases in serum uric acid have been observed in clinical trials, data on serum uric acid levels and the initiation of anti-gout therapy in real-world clinical practice remain limited.

Revised abstract text (page 2, line 5-7)

Clinical trials have shown that bempedoic acid increases serum uric acid levels, but the implications of this effect in real-world clinical practice, including the need for anti-gout treatment, are not well defined.

Comment 2:

Abstract lines 14-17 states "These findings suggest that while bempedoic acid may elevate serum UA, its clinical impact on gout treatment needs appears limited, supporting its continued use in patients requiring further LDL-C lowering while emphasizing the need for monitoring in individuals at risk of hyperuricemia.' Your data doesn't support an emphasis on monitoring uric acid levels in individuals taking bempedoic acid. Further, the guidance in the label for bempedoic acid (Nexletol or Nexlizet) suggests to assess serum acid when clinically indicated. The data supporting anti-gout medication use not being different than the ezetimibe cohort suggests that the serum acid increases weren't leading to differences in treatment between the two groups. The text should be updated to reflect the data.

Response:

We thank the reviewer for this thoughtful comment and agree that our original wording overstated the clinical implications of our findings. While bempedoic acid use was associated with an increased risk of biochemical hyperuricemia, we did not observe a corresponding increase in the initiation of anti-gout therapy compared with ezetimibe, suggesting limited impact on gout-related treatment needs in routine clinical practice.

We have therefore revised the abstract to remove language implying routine uric acid monitoring and to align our conclusions with both the observed data and current prescribing guidance, which recommends assessment of serum uric acid when clinically indicated.

Revised abstract (page 2, line 23-26)

In real-world clinical practice, bempedoic acid use is associated with a higher incidence of laboratory-defined hyperuricemia compared with ezetimibe, but this increase does not translate into greater use of anti-gout medications. These findings support the continued use of bempedoic acid for additional LDL-C lowering, with awareness of its effect on serum uric acid.

Comment 3:

Introduction, page 4, lines 10-16: bempedoic acid is also approved in the US, EU and other countries to lower cardiovascular risk. This should be incorporated into the text.

Response:

We thank the reviewer for this important suggestion. We agree that bempedoic acid has received regulatory approval not only for LDL-C lowering but also for cardiovascular risk reduction in multiple regions. We have revised the Introduction to incorporate this indication and to better reflect the current clinical use of bempedoic acid.

Revised text (page 4, line 10-14)

Bempedoic acid, an oral adenosine triphosphate–citrate lyase inhibitor, is approved in the United States, the European Union, and other regions for use in adults with primary hyperlipidemia, including those with or without atherosclerotic cardiovascular disease (ASCVD) or heterozygous familial hypercholesterolemia (HeFH), to lower low-density lipoprotein cholesterol (LDL-C) and reduce cardiovascular risk.

Comment 4:

Plain Language Summary, Lines 8-9 which state "These findings may help doctors choose cholesterol treatments for patients who are at risk of gout" should be updated to reflect the data. The data doesn't help doctors choose when treatment for patients at risk of gout. Please see other comments and update to reflect the data from this analysis.

Response:

We thank the reviewer for this helpful comment and agree that the original wording overstated the clinical implications of our findings. Our analysis was not designed to guide treatment selection for patients at risk of gout, but rather to evaluate real-world changes in serum uric acid levels and the use of anti-gout therapy associated with bempedoic acid. We have revised the Plain Language Summary to remove language suggesting treatment selection and to more accurately reflect the observational nature and findings of the study.

Revised Plain Language Summary text (page 3, line 9-10)

These findings provide real-world information on how bempedoic acid affects uric acid levels and the need for gout treatment in clinical practice.

Comment 5:

Introduction, page 4, line 27: "However, the clinical relevance of this effect is not yet understood." The clinical relevance of the increase in uric acid is known from clinical trials- it is associated with ~1% increased risk of gout compared to placebo. What is not known is the increase in serum uric acid in clinical practice and the use of anti-gout medications. This sentence needs to be adjusted according. Page 5, Lines 1-9 which is new text is better written and repetitive of the text on page 4. This should be adjusted accordingly.

Response:

We thank the reviewer for this important clarification. We agree that the clinical relevance of bempedoic acid–associated increases in serum uric acid has been demonstrated in randomized clinical trials, including a modestly increased risk of gout compared with placebo. The remaining knowledge gap pertains to the magnitude and clinical consequences of uric acid changes in routine clinical practice, particularly with respect to the use of anti-gout medications.

We have revised the sentence on page 4 to reflect this distinction and have edited the newly added text on page 5 to avoid redundancy by consolidating overlapping content into a single, coherent description of the real-world evidence gap.

Revised text (page 4, line 26 to page 5, line 6)

Bempedoic acid can increase serum UA by inhibiting organic anion transporter 2 (OAT2) in proximal tubules, thereby reducing urate clearance, whereas ezetimibe does not affect OAT2 activity or urate metabolism. Given the increasing use of bempedoic acid as a statin-sparing agent, especially among older adults with multiple comorbidities, characterizing its metabolic side effect profile is essential for informed clinical decision-making. Although randomized trials have quantified the biochemical impact of bempedoic acid on serum UA, including CLEAR Harmony and CLEAR Outcomes, bempedoic acid was associated with a mean serum UA increase of approximately 0.76 mg/dL and a relatively increased risk of gout compared with placebo (3.1% vs. 2.1%). These findings highlight the potential for urate-related adverse events; however, trial participants were highly selected and closely monitored, limiting the applicability of these results to routine clinical practice. Although bempedoic acid is known to increase serum UA levels, the clinical implications of this effect in real-world settings remain uncertain.

Comment 6:

Study Design, page 6: repetition in lines 2-4 with 11-13. This needs to be addressed.

Response:

We thank the reviewer for noting this redundancy. We have revised the Study Design section to eliminate repetitive text by consolidating the overlapping descriptions into a single, concise statement.

Comment 7:

Discussion, page 12, line 24: I have not seen anything to suggest the hyperuricemia events in the clinical program were adjudicated. They were investigator-reported adverse events based on the publications. Please adjust wording accordingly.

Response:

We thank the reviewer for this important clarification and agree that hyperuricemia and gout events reported in the bempedoic acid clinical program were using recorded serum uric acid values and medication records rather than formally adjudicated outcomes. We have revised the Discussion to correct the wording accordingly.

Revised text in discussion (page 12 line 26-28)

This discrepancy is likely explained by the different definitions of outcomes. That is, we defined hyperuricemia using recorded serum uric acid values and medication records, whereas the CLEAR study used investigator-reported adverse events.

Comment 8:

Discussion page 13, lines 28-30: bempedoic acid has been reported to reduce median levels of hsCRP between 20-30%. The data should be stated with supporting publications.

Response:

We thank the reviewer for this helpful suggestion. We agree that the effect of bempedoic acid on high-sensitivity C-reactive protein (hsCRP) has been well described in prior clinical trials. We have revised the Discussion to state the reported magnitude of hsCRP reduction and have added appropriate supporting references.

Revised text (page 13, line 29-30)

in prior randomized clinical trials, bempedoic acid has been shown to reduce median high-sensitivity C-reactive protein levels by approximately 20–30%, suggesting potential anti-inflammatory effects beyond LDL-C lowering. [7, 8, 10]

cited with reference 7, 8, 10

7. Mach F, Koskinas KC, Roeters van Lennep JE, Tokgözoğlu L, Badimon L, Baigent C, Benn M, Binder CJ, Catapano AL, De Backer GG, Delgado V, Fabin N, Ference BA, Graham IM, Landmesser U, Laufs U, Mihaylova B, Nordestgaard BG, Richter DJ, Sabatine MS; ESC/EAS Scientific Document Group. 2025 Focused Update of the 2019 ESC/EAS Guidelines for the management of dyslipidaemias. Eur Heart J. 2025 Nov 7;46(42):4359-4378.
8. Ray, K. K. *et al.* Safety and Efficacy of Bempedoic Acid to Reduce LDL Cholesterol. *New England Journal of Medicine* **380**, 1022–1032 (2019).

10. Nissen, S. E. *et al.* Bempedoic Acid and Cardiovascular Outcomes in Statin-Intolerant Patients. *New England Journal of Medicine* **388**, 1353–1364 (2023).

Comment 9:

Strengths and Limitations: I think you need to clearly indicate as an limitation that the incidence of the anti-gout medications cannot be construed as gout per se as patients may not have treated gout, may receive treatment outside of this claims database, etc. Did you look at events of gout per se?

Response:

We thank the reviewer for this important point and agree that initiation of anti-gout medication should not be interpreted as a definitive diagnosis of gout. Anti-gout therapy use may reflect a range of clinical scenarios, including asymptomatic hyperuricemia, treatment initiated outside of the TriNetX network, or incomplete capture of gout diagnoses.

In this analysis, we did not directly assess adjudicated gout events; instead, initiation of anti-gout therapy was used as a pragmatic real-world proxy for clinically significant hyperuricemia requiring treatment. We have now explicitly acknowledged this limitation in the Strengths and Limitations section.

Revised text (page 15, line 8-14)

In addition, initiation of anti-gout therapy was used as a proxy for clinically significant hyperuricemia and should not be interpreted as a definitive diagnosis of gout. Patients may have experienced gout without receiving pharmacologic treatment, may have received treatment outside of the captured healthcare network, or may have been prescribed anti-gout medications for indications other than acute gout. In addition, gout events were not directly adjudicated or analyzed in this study, potentially leading to an underestimation of clinically symptomatic events.

Comment 10:

Table 3: CRP is not normally distributed and should be reported in median with IQR.

Response:

We thank the reviewer for this important point and agree that C-reactive protein is typically non-normally distributed and is ideally reported as median with interquartile range. We attempted to report CRP using median (IQR); however, the TriNetX analytics platform provides only aggregated summary statistics and does not consistently disclose medians or interquartile ranges for laboratory variables across time points. Consequently, CRP values are presented as mean ± standard deviation, consistent with the reporting structure of the TriNetX database.

In our manuscript, CRP is presented descriptively to characterize longitudinal laboratory patterns rather than to support causal inference; the primary study conclusions rely on time-to-event endpoints and treatment initiation outcomes that do not depend on CRP distributional assumptions. Additionally, this limitation of TriNetX laboratory data reporting has been previously described, including that the platform returns aggregated summary laboratory results and is not optimized for detailed distributional metrics such as IQRs (Wang et al., *Biochemia Medica*, 2025; TriNetX LLC methodological documentation). We have clarified this limitation in the Strengths and Limitations sections of the revised manuscript.

Revised text in limitations (page 15, line 20-23)

CRP was reported as mean ± standard deviation rather than median (interquartile range), as the TriNetX analytics platform does not consistently provide distributional metrics beyond summary statistics for laboratory variables at each time point. This constraint is consistent with prior analyses of TriNetX laboratory data reporting.[24]

We also add references and labelling as number 24

Wang et al., 2025 — Laboratory data limitations with TriNetX

Wang J, Tsai KW, Lu KC, Lu CL. Analyzing clinical laboratory data outcomes in retrospective cohort studies using TriNetX. Biochemia Medica. 2025;35(3):030502. This paper specifically states that TriNetX only returns the most recent laboratory test result in a given period and that the platform is not optimized for detailed interval laboratory analyses, explaining platform limitations for lab outcomes.

Response to Editors Comments

Thank you very much for your positive decision and for the opportunity to revise our manuscript. We have carefully addressed all requested amendments as detailed below

To the Editor:

The authors sincerely thank the editor for the thoughtful and constructive comments. Each point has been carefully addressed below, and the manuscript has been revised accordingly to improve clarity, accuracy, and transparency.

Comment 1:

Please adjust the Results section of your Abstract to be in the present tense.

Response:

The Results section of the Abstract has been revised to present tense.

Revised result text:

During 12 months of follow-up, hyperuricemia occurs more frequently in patients treated with bempedoic acid than in those receiving ezetimibe (hazard ratio [HR] 1.94; 95% confidence interval [CI] 1.58–2.37; p = 0.008). In contrast, initiation of anti-gout therapy does not differ between groups (HR 1.06; 95% CI 0.86–1.29; p = 0.59). Longitudinal changes in lipid parameters, inflammatory markers, and glycemic measures are generally similar between groups.

Comment 2:

Please add additional information in your Data Sources section of your Methods indicating how access was gained to the TriNetX database. Additionally, we ask that you clarify the wording of the IRB approval - did the Western IRB grant a waiver of the need for informed consent to the present study, or TriNetX in general?

Response:

The Data Sources section has been expanded to clarify institutional access to TriNetX and the IRB exemption determination by the Western Institutional Review Board.

Revised data sources

The retrospective cohort study used de-identified electronic health record data from the TriNetX Research Network (TriNetX LLC, Cambridge, MA, USA), a federated research

platform comprising electronic medical records from approximately 131 million patients across 71 healthcare organizations. Data were accessed on 3 May 2025. The National Cheng Kung University Hospital has institutional access to the TriNetX platform through a data use agreement with TriNetX LLC. All data available through TriNetX are de-identified in accordance with the Health Insurance Portability and Accountability Act (HIPAA) Privacy Rule.

The use of de-identified TriNetX data was reviewed by the Institutional Review Board of National Cheng Kung University Hospital and determined to be exempt from full review (Approval No. B-ER-114-232). The TriNetX platform itself operates under a waiver of informed consent granted by the Western Institutional Review Board (WIRB), as all data are de-identified. The study was carried out in accordance with the Strengthening the Reporting of Observational Studies in Epidemiology (STROBE) guidelines.

Comment 3:

You must add a Data Availability statement following your Conclusions, indicating how your data may be shared with readers. Also explain in this section how a user can gain access to the TriNetX dataset.

Response:

A Data Availability statement has been added following the Conclusions and before References section.

Revised data availability statement:

The data that support the findings of this study are available from the TriNetX Research Network (TriNetX LLC), but restrictions apply to the availability of these data, which were used under license for the current study and are not publicly available. Researchers may obtain access to the TriNetX platform through institutional subscription and approval by TriNetX.

Comment 4:

Tables 1 and 3 are larger than our formatting currently permits. We suggest that you upload Table 3 as an Excel file under Supplementary Data, and that Table 1 be either moved to

the Supplementary Information, or broken down into multiple tables that can fit within a standard A4 page.

Response:

Table 3 has been uploaded as Supplementary table 4 (Excel format), and Table 1 has been moved to the Supplementary table 3 Information to comply with formatting requirements.

To the assistant editor:

We sincerely thank the editor for the thoughtful and constructive comments. Each point has been carefully addressed below, and the manuscript has been revised accordingly to enhance clarity, accuracy, and transparency.

Comment 1:

Please note that the exact p value should be provided, when possible, in the legends of figures and tables.

Response:

Exact p values have been added to all relevant figure and table legends.

Revised table 1:

Table 1. Comparative incidence of hyperuricemia and the initiation of antigout therapy.

Outcome	Treatment Group	Patients (n)	Events (n)	Hazard Ratio (95% CI)	p value
Hyperuricemia	BA	7,675	266	1.94 (1.58 - 2.37)	p=0.008
	EZ	7,675	149		
Initiation of Treatment for Hyperuricemia	BA	7,675	187	1.06 (0.86 - 1.29)	p=0.59
	EZ	7,675	190		

Note: BA, bempedoic acid and EZ, ezetimibe.
